# Glucose uptake in *Azotobacter vinelandii* occurs through a GluP transporter that is under the control of the CbrA/CbrB and Hfq-Crc systems

**DOI:** 10.1038/s41598-017-00980-5

**Published:** 2017-04-12

**Authors:** Elva Quiroz-Rocha, Renata Moreno, Armando Hernández-Ortíz, Juan Carlos Fragoso-Jiménez, Luis Felipe Muriel-Millán, Josefina Guzmán, Guadalupe Espín, Fernando Rojo, Cinthia Núñez

**Affiliations:** 1 Departamento de Microbiología Molecular, Instituto de Biotecnología, Universidad Nacional Autónoma de México, Av. Universidad 2001, Col Chamilpa, Cuernavaca, 62210 Morelos México; 2 Departamento de Biotecnología Microbiana, Centro Nacional de Biotecnología, CSIC, Darwin 3, Cantoblanco, 28049 Madrid, Spain; 3 Departamento de Ingeniería Celular y Biocatálisis, Instituto de Biotecnología, Universidad Nacional Autónoma de México, Av. Universidad 2001, Col Chamilpa, Cuernavaca, 62210 Morelos México

## Abstract

*Azotobacter vinelandii*, a strict aerobic, nitrogen fixing bacterium in the *Pseudomonadaceae* family, exhibits a preferential use of acetate over glucose as a carbon source. In this study, we show that GluP (Avin04150), annotated as an H^+^-coupled glucose-galactose symporter, is the glucose transporter in *A*. *vinelandii*. This protein, which is widely distributed in bacteria and archaea, is uncommon in *Pseudomonas* species. We found that expression of *gluP* was under catabolite repression control thorugh the CbrA/CbrB and Crc/Hfq regulatory systems, which were functionally conserved between *A*. *vinelandii* and *Pseudomonas* species. While the histidine kinase CbrA was essential for glucose utilization, over-expression of the Crc protein arrested cell growth when glucose was the sole carbon source. Crc and Hfq proteins from either *A*. *vinelandii* or *P*. *putida* could form a stable complex with an RNA A-rich Hfq-binding motif present in the leader region of *gluP* mRNA. Moreover, in *P*. *putida*, the *gluP* A-rich Hfq-binding motif was functional and promoted translational inhibition of a *lacZ* reporter gene. The fact that *gluP* is not widely distributed in the *Pseudomonas* genus but is under control of the CbrA/CbrB and Crc/Hfq systems demonstrates the relevance of these systems in regulating metabolism in the *Pseudomonadaceae* family.

## Introduction

*Azotobacter vinelandii* is a gamma proteobacterium belonging to the *Pseudomonadaceae* family^1^. Although *A*. *vinelandii* is phylogenetically related to *Pseudomonas* species, it possesses distinctive features that clearly separates it from the genus *Pseudomonas*, such as its capacity to differentiate and form dormant cysts that are resistant to drought, its strict aerobic metabolism and its capacity to fix nitrogen under aerobic conditions^2, 3^. *A*. *vinelandii* produces two polymers of biotechnological importance, the exo-polysaccharide alginate and the intracellular polyester polyhydroxybutyrate^4^. *A*. *vinelandii* can use many carbohydrates, alcohols and salts of organic acids for growth; however, it is unable to grow using amino acids as the sole carbon source^5^. Similar to *Pseudomonas* spp., *A*. *vinelandii* does not have a functional glycolytic pathway, but instead relies on the Entner-Doudoroff pathway (EDP) for glucose utilization^6^. This bacterium exhibited diauxic growth when grown in a medium containing both acetate and glucose^7–9^. In this condition, acetate was used as the primary carbon source. Once the acetate was exhausted, glucose uptake was initiated, indicating that acetate repressed the synthesis of the glucose transport system. The tricarboxylic acid intermediates citrate, isocitrate and 2-oxo-glutarate also inhibited glucose utilization^9^, suggesting the existence of a carbon catabolite repression (CCR) process in *A*. *vinelandii* similar to that in *Pseudomonas* species.

CCR is a global regulatory system that allows the selective assimilation of a preferred compound among a mixture of several potential carbon sources. Unlike *Escherichia coli* and *Bacillus subtilis*, the preferred carbon sources for bacteria in the genus *Pseudomonas* are some organic acids and amino acids rather than glucose^10^. In *Pseudomonas* species the process of CCR is elicited mainly through a regulatory system based on the Crc and Hfq proteins and one or more small RNAs (sRNAs) of the CrcZ, CrcY or CrcX family that antagonize the effect of these regulatory proteins^11–14^. A two-component system, composed of the histidine kinase (HK) CbrA and the response regulator (RR) CbrB, heads this regulatory pathway by directly activating the transcription of sRNAs from RpoN-dependent promoters^14, 15^. The Crc and Hfq proteins play a central role in CCR in *Pseudomonas* spp., promoting the inhibition of translation of RNAs containing an AAnAAnAA motif, called the CA motif (for Catabolite Activity), which is close to the translation initiation site^14, 16–19^. The RNA chaperone Hfq recognizes and binds these A-rich motifs, the role of Crc being to stabilize the ribonucleoprotein complexes formed^12, 13, 20^. For this reason, the CA motifs are also known as A-rich Hfq-binding motif. As noted above, glucose is not the preferred carbon source for *Pseudomonas*. In fact, Crc and Hfq inhibit the expression of the OprB1 porin for the uptake of glucose, of the inner membrane glucose transporter GtsABC, and of several genes required for glucose assimilation in *P*. *putida* when grown in a rich medium^21^.

In the present study, we investigated the regulation of glucose uptake in *A*. *vinelandii* and the role of the CbrA/CbrB and Crc/Hfq systems in the CCR process. Analysis of the genome sequence revealed that *A*. *vinelandii* lacks the inner membrane glucose transporter GtsABC that is present in *Pseudomonas* spp. Our results indicated that the gene *gluP* encodes the glucose transporter and that translation of the *gluP* mRNA is under catabolite repression control thorugh the CbrA/CbrB and Crc/Hfq regulatory systems in *A*. *vinelandii*. The *gluP* A-rich Hfq-binding motif was functional when introduced into *P*. *putida* as its presence promoted translational inhibition of a *lacZ* reporter gene.

## Results

### The histidine kinase CbrA is necessary for the utilization of glucose

Previous studies demonstrated the preferential use of acetate over glucose in the *A*. *vinelandii* strain OP^8, 9^, a derivative of the O strain that is unable to produce the exo-polysaccharide alginate^2^. However, the underlying mechanism of this catabolic repression was unknown. In a random miniTn*5* mutant bank derived from the wild-type strain AEIV, we identified mutant GG15 by its alginate-over-producing phenotype on plates of minimal Burk’s-sucrose medium. This mutant carries the miniTn*5* insertion within codons 660 and 661 of a gene that is similar to *cbrA*, which encodes an HK that forms part of the CbrA/CbrB two-component system present in *Pseudomonas* spp. As explained in the Introduction, the CbrA/CbrB regulatory system plays a key role in *Pseudomonas* catabolite repression together with Crc and Hfq proteins, and the CrcZ/CrcY sRNAs.

**Figure 1:**
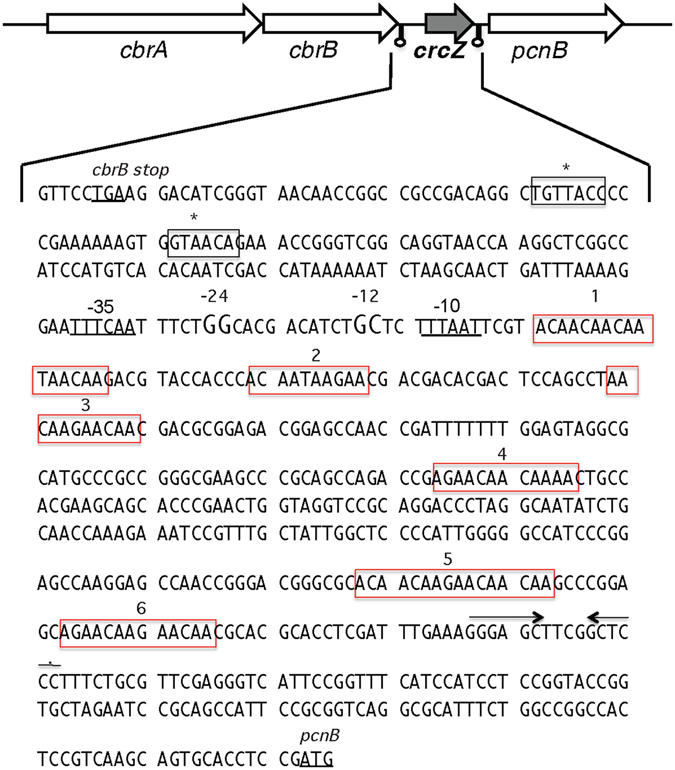
Genome context and nucleotide sequence of the *A*. *vinelandii* CrcZ sRNA. Terminators are indicated by small stem-loops. The six CrcZ A-rich Hfq-binding motifs are indicated by red boxes. The −12 and −24 regions of the predicted RpoN promoter and putative sequences recognized by CbrB (black boxes) are indicated. The predicted −10 and −35 regions of an RpoD promoter are underlined. Inverted repeats at the end of CrcZ are denoted by solid arrows.

Genes encoding all components of the CbrA/CbrB and Crc/Hfq systems are present in the *A*. *vinelandii* genome. A *cbrB* gene (Avin42680), encoding the CbrB cognated RR, and the gene for the sRNA CrcZ, are located immediately downstream of *cbrA* (Avin42670) (Fig. 1). The predicted CbrA and CbrB proteins are highly similar (>77% identity) to their homologues from *P*. *aeruginosa* and *P*. *putida*. An *in-silico* analysis identified a putative σ^70^ promoter upstream of the *cbrB* gene, which has also been identified in *P*. *aeruginosa* ^22^. The *A*. *vinelandii* genome also contains genes for the sRNAs CrcZ and CrcY^11^. The CrcZ sRNA shows 68% identity to the *P*. *putida* and *P*. *aeruginosa* CrcZ and contains six putative A-rich Hfq-binding motifs (AANAANAA) (Fig. 1). The predicted secondary structure shows that these A-rich motifs are located on the loops (Supplementary Fig. S1). The *A*. *vinelandii crcY* gene was located in the intergenic region in between Avin04820 and Avin04790 ORFs, which encode hypothetical proteins (Supplementary Fig. S2). CrcY contains five putative A-rich Hfq-binding motifs, and four of them are predicted to be located on the loops (Supplementary Fig. S3).

Genes *crc* (Avin02870) and *hfq* (Avin07540) were also identified in the *A*. *vinelandii* genome. The Crc and Hfq proteins showed 88 and 93% identity, respectively, to their *P*. *aeruginosa* homologues. The genes are in regions highly conserved among several *Pseudomonas* species. A BLAST search indicated that *crcZ*, *crcY*, *crc* and the *hfq* genes are present in the genome of the *A*. *vinelandii* strain CA-6 and in *Azotobacter chroococcum* within similar genomic contexts.

As mentioned above, the CbrA-null strain GG15 over-produced alginate when cultivated on plates of minimal Burk’s-sucrose medium. In fact, the GG15 mutant showed five-times higher alginate levels than the wild type strain AEIV^23^. Since this polymer is synthesized from fructose-6-phosphate it competes for the available carbon source affecting cell growth^24^. Thus, the alginate phenotype of mutant GG15 would prevent a reliable assessment of the CbrA role on the growth on different carbon sources. For this reason, the effect of the HK CbrA on the CCR process was evaluated in an *alg*-negative genetic background. We used strain AEalgD, a derivative of strain AEIV that carries an *algD*::Km mutation, and mutant AH1, an AEalgD derivative carrying a *cbrA*::miniTn*5* mutation.

**Figure 2:**
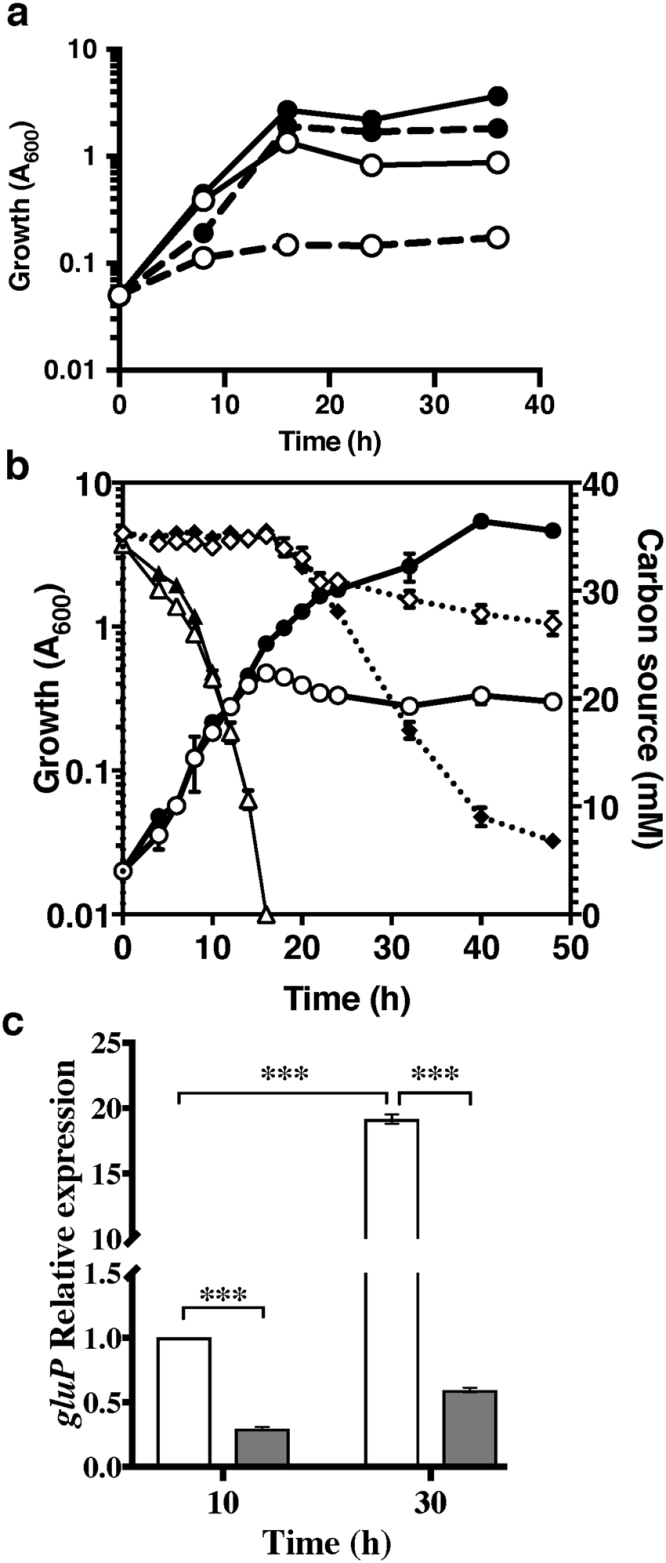
The HK CbrA is necessary for glucose utilization. (**a**) Growth kinetics of the reference strain AEalgD (*algD*::Km) (closed symbols) and its derivative mutant AH1 (*cbrA*::miniTn*5*) (open symbols) in liquid Burk’s minimal medium amended with 50 mM glucose (dashed lines) or 2% sucrose (solid lines). (**b**) Growth kinetics (circles), and acetate (triangles) or glucose (diamonds) consumption during the culture of the reference strain AEalgD (*algD*::Km) (closed symbols) or its derivative mutant AH1 (*cbrA*::miniTn5) (open symbols) in Burk’s minimal medium supplemented with both 30 mM glucose and 30 mM acetate as carbon sources. (**c**) *gluP* mRNA levels determined by qRT-PCR analysis in strain AEalgD (white columns) and in mutant AH1 (grey columns) in the diauxic growth of panel (**b**). Total RNA was extracted from cells at the indicated times. The bars of standard deviation from three independent experiments are shown. Significant differences were analysed by *t*-test. Statistical significance is indicated (***p < 0.001).

As shown in Fig. 2a, both AEalgD and AH1 strains grew with sucrose as the sole carbon source. However, strain AH1 was unable to grow on glucose, indicating that the CbrA HK was necessary for the utilization of this compound in *A*. *vinelandii*. Figure 2b shows that the assimilation of glucose did not occur in the presence of acetate in the AEalgD strain, which was consistent with previous reports indicating diauxic growth in *A*. *vinelandii* in medium supplemented with acetate and glucose. However, after acetate consumption, glucose utilization was initiated. In contrast, the *cbrA* mutant AH1 grew on acetate plus glucose, but glucose assimilation was abrogated and growth ceased as soon as acetate was consumed (Fig. 2b). The inability to use glucose was also observed for an AEalgD derivative *cbrA*::Sp mutant constructed by reverse genetics (data not shown). These results indicate that the HK CbrA was involved in the control of CCR in *A*. *vinelandii*. Because the CbrA/CbrB system is needed in *P*. *putida* and *P*. *aeruginosa* for the synthesis of the CrcZ/CrcY sRNAs, which in turn antagonize the effects of Crc/Hfq^13, 20, 25^, the lack of CbrA in *A*. *vinelandii* could be generating strong Crc/Hfq-dependent repression. We next analysed this possibility.

### The two-component system CbrA/B was necessary for the transcription of the sRNAs CrcZ and CrcY

**Figure 3:**
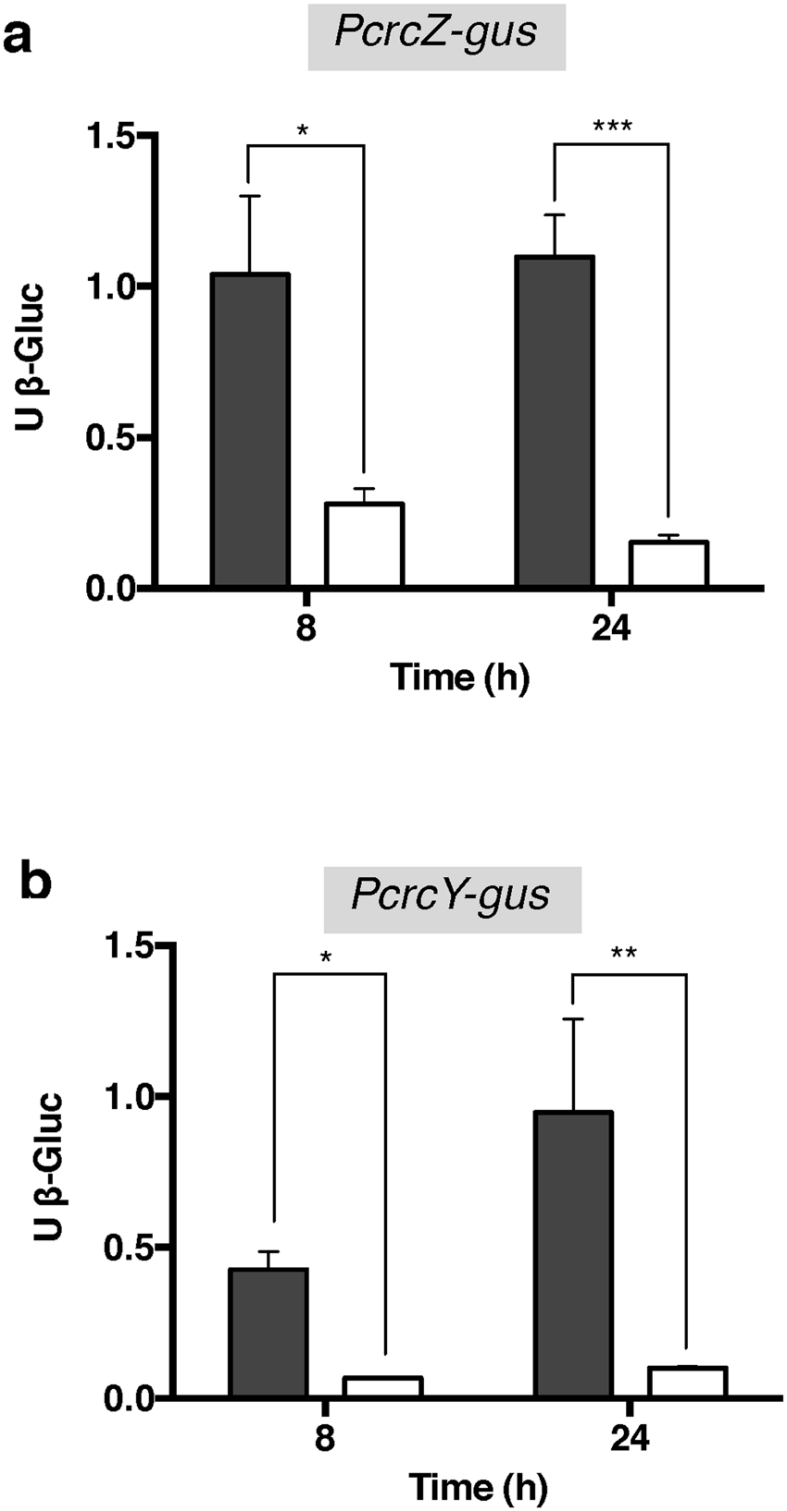
Effect of the HK CbrA on *crcZ* and *crcY* gene expression. Expression of *crcZ* and *crcY* genes was assessed by *PcrcZ*-*gusA* (**a**) and *PcrcY*-*gusA* (**b**) transcriptional fusions, respectively. These transcriptional fusions were tested in wild type and *cbrA* genetic backgrounds. The strains used were: AE-Zgus (wild type; black bars) and CbrA-Zgus (*cbrA*::Sp; white bars) (**a**); AE-Ygus (wild type; black bars) and CbrA-Ygus (*cbrA*::Sp; white bars) (**b**). Cultures were developed in minimal Burk’s-sucrose medium. Aliquots were taken at the indicated times and ß-glucuronidase activity was measured. The bars of standard deviation from three independent experiments are shown. Significant differences were analysed by *t*-test. Statistical significance is indicated (*p < 0.05, **p < 0.01 or ***p < 0.001).

To determine whether expression of the sRNAs CrcZ and CrcY in *A*. *vinelandii* was under the control of the two-component system CbrA/CbrB, transcriptional fusions of the regulatory regions of *crcZ* and *crcY* with the reporter gene *gusA* were constructed (*PcrcZ-gusA* and *PcrcY-gusA*, respectively). Derivatives of the wild type strain AEIV and mutant *cbrA* carrying these gene fusions were cultured in Burk’s-sucrose medium, and the activity of ß-glucuronidase was measured at the exponential (8 h) and stationary (24 h) phases of growth. As shown in Fig. 3, transcription of both sRNAs was dependent on the HK CbrA as in the *cbrA* mutant the activity of the *PcrcY-gusA* and *PcrcZ-gusA* transcriptional fusions was reduced at least four-fold during the exponential and stationary growth phases. Analysis of the CrcZ regulatory region allowed the identification of conserved residues characteristic of RpoN dependent promoters located at 13 (−12) and 25 (−24) nt upstream of the first A-rich Hfq-binding motif. Additionally, putative binding sites for the RR CbrB at positions −125 and −145 relative to the first A-rich Hfq-binding motif were identified (Fig. 1a). The regulatory region of *crcY* also showed conserved sequences for an RpoN-dependent promoter as well as sequences recognized by the RR CbrB at similar positions (Supplementary Fig. S2). Together, these results imply that the two-component CbrA/CbrB system activates CrcZ and CrcY transcription, as has been described in several *Pseudomonas* species.

### The sRNA CrcZ can bind to the Hfq protein and Crc facilitates this interaction

**Figure 4:**
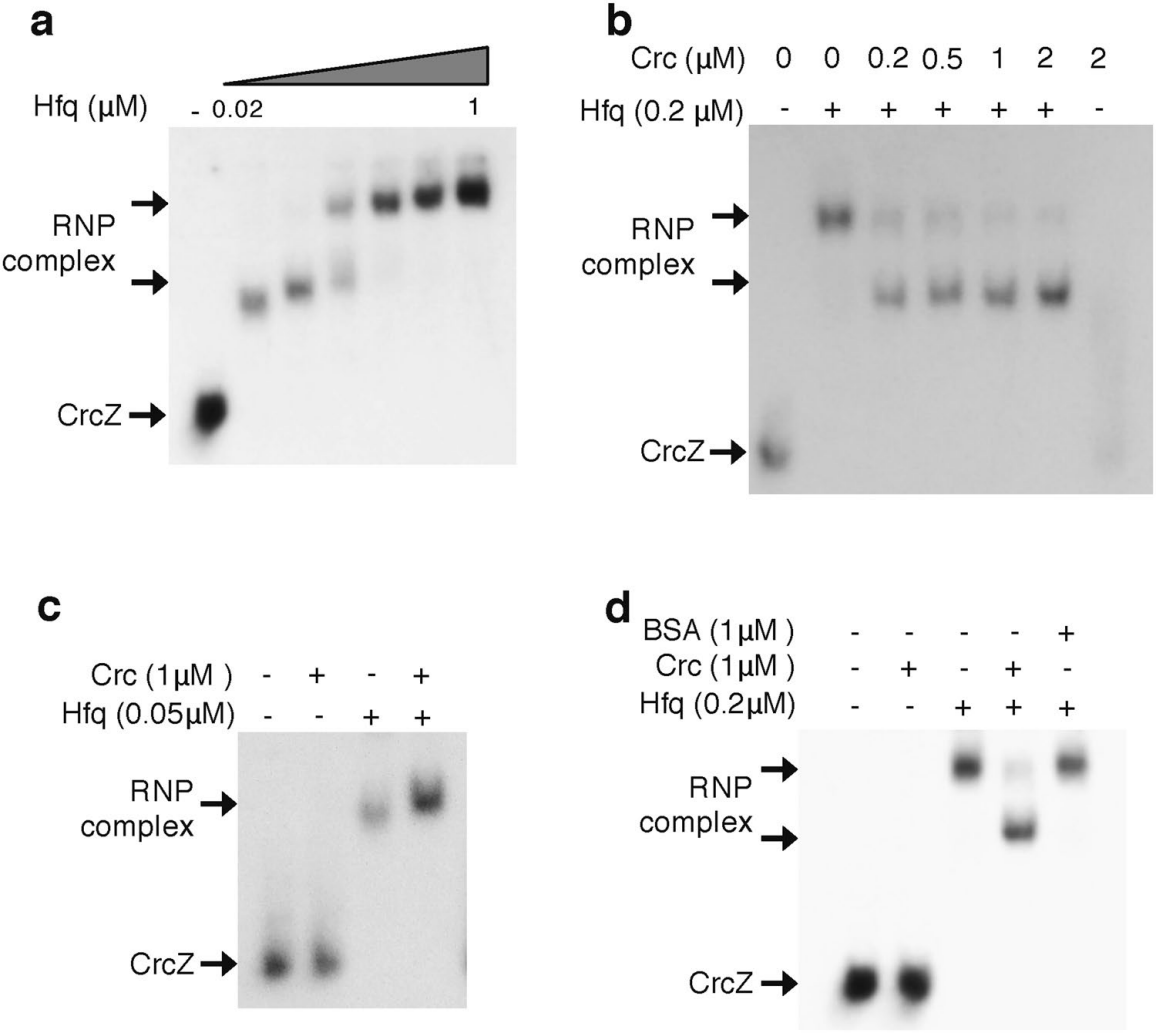
Hfq-Crc proteins form a stable ribonucleoprotein complex with CrcZ. Ribonucleoprotein complexes formed in the presence of the sRNA CrcZ and increasing concentrations of Hfq (0, 0.02, 0.05, 0.1, 0.2, 0.5 and 1 µM) (**a**) or in the presence of both Crc and Hfq (**b** and **c**). In (**d**) Crc was substituted by 1 µM of bovine serum albumin (BSA). Crc and Hfq were added at the indicated concentrations (expressed as monomers and hexamers respectively). RNA and protein-RNA complexes were resolved in a non-denaturing polyacrylamide gel. The position of free RNA, and of the ribonucleoprotein (RNP) complexes detected, is indicated.

We next investigated the ability of CrcZ to form a complex with the purified His-Hfq and Crc proteins from *A*. *vinelandii* using RNA electrophoretic-mobility shift assays (EMSAs). To avoid *E*. *coli* Hfq contaminations His-Hfq and Crc proteins were purified from the Hfq-null *E*. *coli* expression strain MG1655^12^. Radioactively labelled CrcZ was synthesized by *in vitro* transcription. Binding reactions were performed using the protocols reported for *P*. *putida*, in the presence of an excess of tRNA allowing specific binding of the Hfq and Crc proteins^16, 18^. Hfq generated a stable complex with CrcZ at concentrations as low as 0.02 μM (Fig. 4a). As the concentration of Hfq increased, the formed complex showed lower mobility consistent with the presence of several A-rich Hfq-binding motifs in CrcZ. This complex also showed increased stability based on the intensity of the shifted band (Fig. 4a). The Crc protein alone was unable to form a stable complex with CrcZ, even at high concentrations (Fig. 4b). However, at 0.2 µM Hfq, the presence of Crc changed the migration pattern of the Hfq-CrcZ complex, which showed a faster electrophoretic mobility, suggesting the formation of a tripartite CrcZ-Hfq-Crc complex (Fig. 4b). At a lower Hfq concentration (0.05 µM), the presence of Crc also stabilized the Hfq-RNA complex formed (Fig. 4c). The presence of Crc but not that of bovine serum albumin (BSA) changed the migration pattern of the shifted band (Fig. 4d), implying that Crc binds to the CrcZ-Hfq complex in a specific manner. Collectively these results indicate that the CbrA/CbrB and Crc-Hfq systems in *A*. *vinelandii* and *Pseudomonas* spp. operate mechanistically in a similar manner.

### Over-expression of Crc in trans diminishes growth on glucose

**Figure 5:**
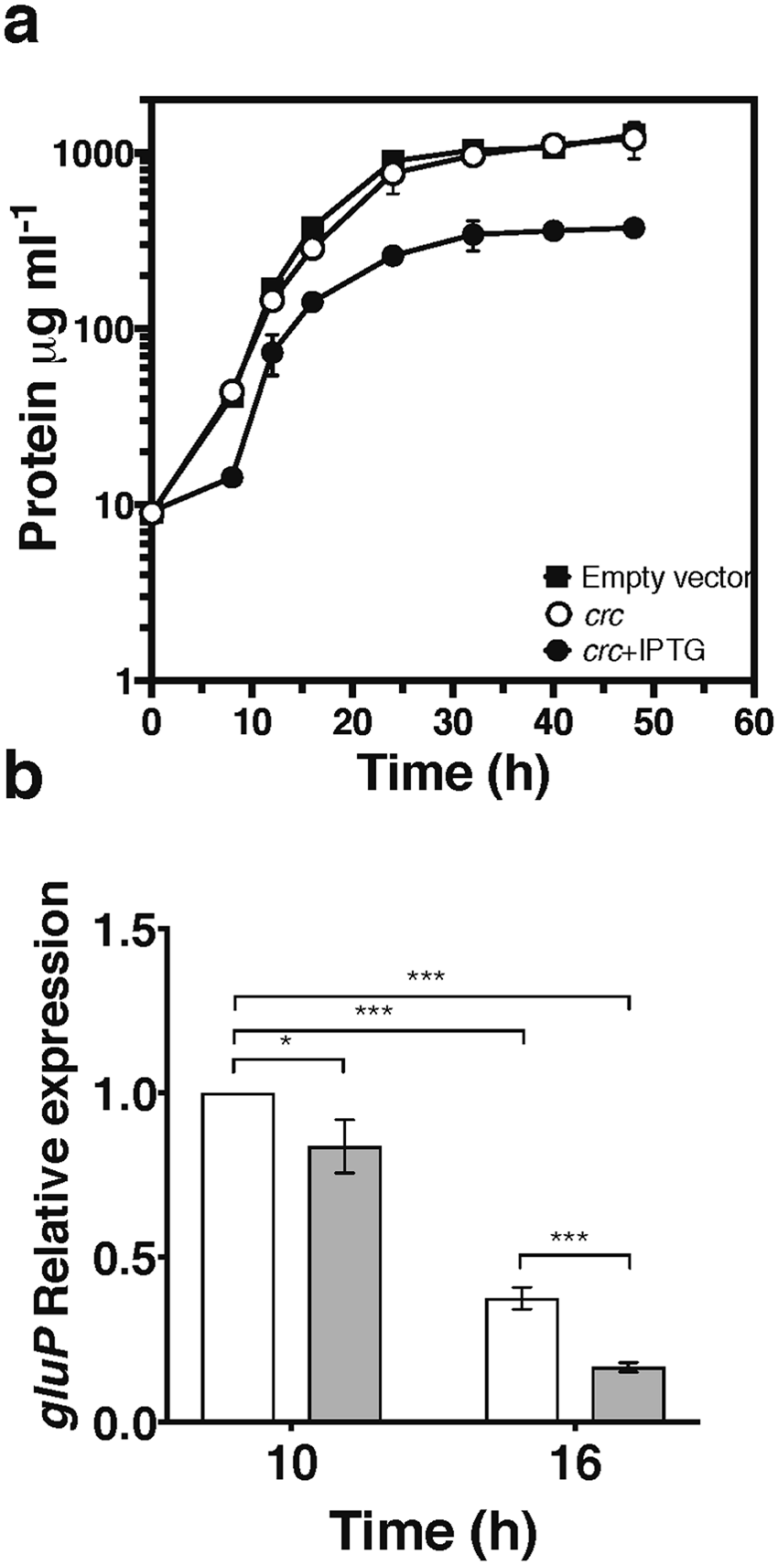
Crc over-expression diminishes growth on glucose. (**a**) Growth kinetics of the wild type *A*. *vinelandii* AEIV strain, harbouring the empty vector pSRK-Km (■) or the pSRK-crc (*crc* ^+^) vector, in the presence (●) or absence (○) of 1 mM IPTG. Cultures were developed in Burk’s minimal medium supplemented with 30 mM glucose (BG) as the sole carbon source and 1.5 µgml^−1^ of kanamycin as a selection marker. 25 ml of Burk’s medium supplemented with 30 mM sucrose were cultured for 18 h; cells were harvested by centrifugation, washed with phosphate buffer 10 mM pH 7.2 and resuspended in the same solution. 400 µg of these cells were used to inoculate 50 ml of BG medium and samples were collected at the indicated times for protein quantification. Cell growth was estimated by determining protein concentration since the production of the exo-polysaccharide alginate by the AEIV strain prevents assessment of growth by optical density. The results represent the averages of the results of three independent experiments, and error bars depict standard deviations. (**b**) Relative expression levels of *gluP* mRNA, quantitated by qRT-PCR analysis, in cells of the wild type AEIV strain carrying the vector pSRK-crc in the absence (white colums) or presence (grey columns) of 1 mM IPTG. The growth conditions were as in panel (**a**). Total RNA was extracted from cells at the indicated times. The bars of standard deviation from three independent experiments are shown. Significant differences were analysed by *t*-test. Statistical significance is indicated (*p < 0.05 or ***p < 0.001).

As shown above, the *cbrA* mutant strain AH1 was unable to use glucose in the presence of acetate. Thus, we explored the possibility that the effect of the CbrA/CbrB system on glucose assimilation was exerted through the Crc system. Efforts to construct strains carrying *crc* or *crcZ* mutations were unsuccessful. The selection of such mutants was conducted using minimal or rich medium supplemented with acetate, succinate, glucose or sucrose as carbon sources, either under diazotrophic conditions or in the presence of fixed nitrogen. This result suggested that the Crc protein and the CrcZ sRNA are necessary for the vegetative growth of *A*. *vinelandii* under laboratory conditions. Therefore, we evaluated the effect of Crc over-expression on glucose assimilation by using plasmid pSRK-crc, a pSRK-Km derivative vector carrying the *A*. *vinelandii crc* gene transcribed from a *lac* promoter induced only in the presence of IPTG^26^. For this purpose we used the wild type strain AEIV because increasing levels of Crc did not affect alginate synthesis (data not shown). Thus, pSRK-crc and the empty vector pSRK-Km were transferred to the AEIV strain, and the ability of the resulting strains to grow with glucose as the sole carbon source was evaluated. As shown in Fig. 5a, the AEIV strain carrying the empty vector exhibited normal growth and reached the stationary phase at 24 h of growth. This behaviour was similar for the AEIV strain carrying the pSRK-crc vector in the absence of IPTG. In the presence of 1 mM IPTG, however, the AEIV strain harbouring the pSRK-crc vector showed a growth lag during the first 10 h; after this time growth was resumed but started to decline at 16 h of culture reaching a maximum protein concentration of 375 µgml^−1^, three-fold lower than the control strain. These data indicated a negative effect of the Crc protein on glucose catabolism.

When cultivated with sucrose, Crc over-expression weakly diminished cell growth at the end of the exponential phase, reaching twofold less protein concentration than the uninduced culture (Supplementary Fig. S4). This result was somewhat expected given the effect that Crc showed on glucose catabolism. In contrast, growth in the presence of preferred carbon sources such as acetate or succinate was not affected (Supplementary Fig. S4). Altogether these results imply that higher levels of Crc simulate strong CCR conditions preventing the utilization of glucose, a secondary carbon source for *A*. *vinelandii*.

### GluP is the glucose transporter in *A. vinelandii*

**Figure 6:**
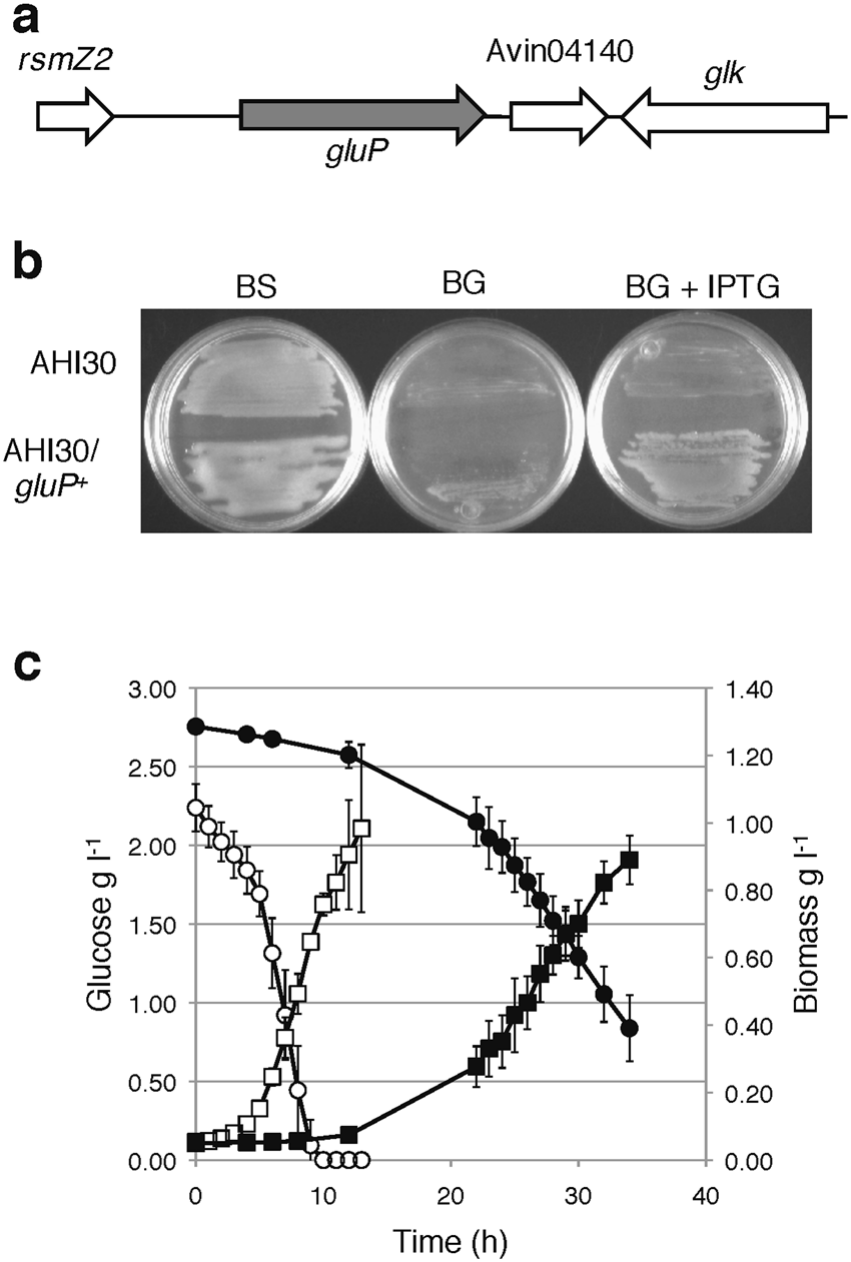
The gene *gluP* of *A*. *vinelandii* encodes a glucose transporter. (**a**) Genomic context of the *A*. *vinelandii gluP* gene. (**b**) Growth of the *A*. *vinelandii glup*::Sp mutant AHI30 and its derivative carrying the pSRK-gluP plasmid (*gluP* ^+^) on plates of solid Burk’s medium supplemented with 2% sucrose (BS) or 50 mM glucose (BG). Where indicated, 1 mM IPTG was added to induce transcription of *gluP* from the *lac* promoter. (**c**) Growth kinetic, measured as cell biomass (g l^−1^) (squares) and glucose consumption (circles) by the *E*. *coli* WHIPC mutant (closed symbols) or its derivative carrying the pSRK-gluP (*gluP* ^+^) plasmid (open symbols), in the presence of 0.1 mM IPTG. Cultures were developed in M9 mineral medium supplemented with 2.5 g l^−1^ of glucose.

Analysis of the *A*. *vinelandii* genome allowed us to identify several genes involved in glucose metabolism and that contain putative A-rich Hfq-binding motifs near the translation start site (Supplementary Table S1). Of interest, the *A*. *vinelandii* genome lacks the typical glucose ABC transporter GtsABC present in *Pseudomonas* spp. but contains the gene Avin04150 (*gluP*), which was annotated as a transporter for glucose and galactose belonging to the Major Facilitator Superfamily of the Fucose-Galactose-Glucose:H^+^ symporter (FGHS) family (Fig. 6a)^27, 28^. The *gluP* gene encodes a 429 amino acid protein predicted to have 12 trans-membrane helices typical of the members of the MFS^29^. This protein showed 48% identity with the glucose-galactose transporter (GluP) from *Brucella abortus* ^30^. Homologues of the *A*. *vinelandii* GluP were also present in *A*. *chroococcum* and *Azotobacter beijerinckii* (showing 90% identity) and in a few *Pseudomonas* species (some of them were associated with plants).

To determine the role of GluP in glucose assimilation, strain AHI30 carrying a *gluP*::Sp mutation was constructed and characterized. As shown in Fig. 6b, this mutant was able to grow on sucrose; however it failed to grow on medium supplemented with glucose as the sole carbon source. Genetic complementation of AHI30 mutant with a wild type copy of *gluP* was conducted. To this end, *gluP* was cloned in the vector pSRK-Km, producing plasmid pSRK-gluP. Growth with glucose as the sole carbon source was restored for the AHI30 mutant harbouring plasmid pSRK-gluP only in the presence of 1 mM IPTG, implying that GluP is necessary for the uptake of this carbohydrate (Fig. 6b).

An additional approach was used to confirm the role of GluP as the glucose transporter in *A*. *vinelandii*. Heterologous genetic complementation of an *E*. *coli* strain devoid of the major glucose transporter systems with the *A*. *vinelandii gluP* gene was conducted. We constructed an *E*. *coli* mutant carrying multiple deletions of genes encoding the general components of the PTS system, the galactose:H^+^ symporter GalP and the Mgl galactose/glucose ABC transporter and named it WHIPC. Plasmids pSRK-Km (control) and pSRK-gluP were transferred to the WHIPC strain and the resulting transconjugants were tested for the ability to grow in M9 mineral medium amended with 2.5 gl^−1^ glucose. As shown in Fig. 6c, growth of the WHIPC mutant occurred at a specific growth rate of 0.12 h^−1^ and a maximum specific rate of glucose consumption of 0.01 g g of dry cell weight (DCW)^−1 ^h^−1^. However, growth of the mutant WHIPC carrying the pSRK-gluP vector in M9 medium supplemented with 2.5 gl^−1^ glucose and 0.1 mM IPTG was clearly improved, exhibiting a specific growth rate of 0.34 h^−1^ and a maximum specific rate of glucose consumption of 0.1 g gDCW^−1 ^h^−1^, which was ten-fold higher compared with the WHIPC strains (Fig. 6c). Several unsuccessful attempts to grow strain WHIPC harbouring the control plasmid pSRK-Km in liquid mineral M9 medium amended with glucose and kanamycin were made, which implied that the vector itself represented a genetic load that prevented cell growth. These results indicated that the *A*. *vinelandii* GluP protein functions as a glucose transporter in *E*. *coli*.

### Expression of gluP is under the control of the CbrA/CbrB and Hfq-Crc systems

Because in *A*. *vinelandii* the catabolism of glucose is prevented as long as acetate is still present in the culture medium, we speculated that expression of the GluP encoding gene would be repressed under conditions of strong catabolite repression (i.e. in the presence of acetate). As expected, *gluP* mRNA levels, assessed by qRT-PCR analyses, were low in the presence of acetate but increased 19-times during glucose catabolism in the AEalgD strain (Fig. 2c). This response was dependent on the HK CbrA as in the *cbrA* mutant AH1 the levels of *gluP* were low throughout the growth curve with respect to the parental strain AEalgD (Fig. 2c).

We next investigated whether the growth inhibition upon Crc over-expression was associated with reduced expression of *gluP*. *gluP* mRNA levels were determined by qRT-PCR at two points of the growth kinetics (6 and 10 h), corresponding to the exponential growth phase of the reference strains. Interestingly, at 10 h Crc over-expression lowered *gluP* mRNA levels by 20% whereas at 16 hr this effect was substantially greater as the levels of *gluP* were reduced by 60%, thus confirming that the Crc protein has a negative effect on the transcript abundace of *gluP* (Fig. 5b). Altogether these results suggested that *gluP* is a direct target of the Hfq-Crc proteins in the process of CCR.

### The *A. vinelandii* Hfq protein binds to the leader of *gluP* and Crc enhances this interaction

**Figure 7:**
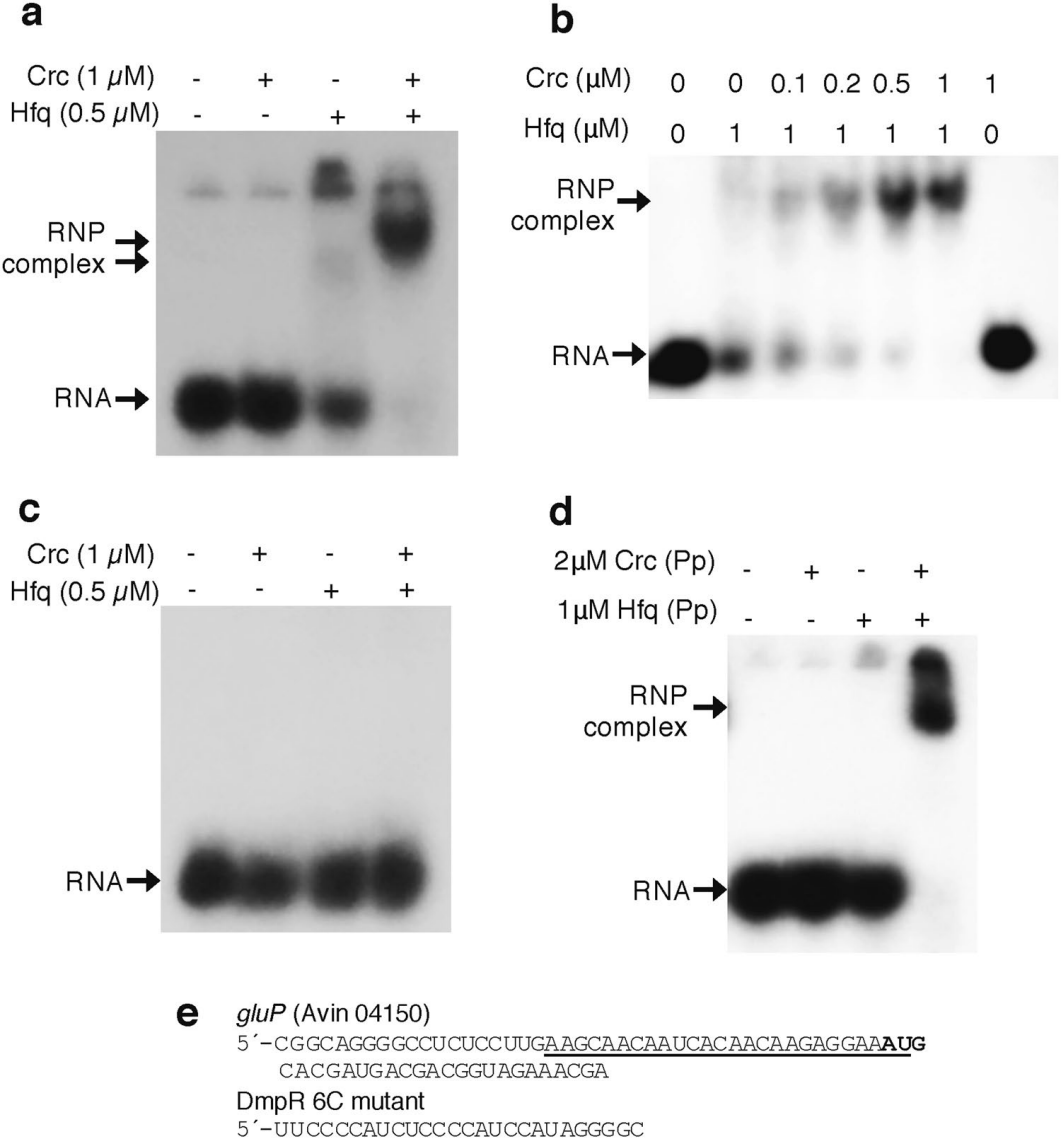
Hfq-Crc proteins form a stable ribonucleoprotein complex with the A-rich Hfq-binding RNA motif of *gluP*. (**a**) Binding of the *A*. *vinelandii* Crc and Hfq proteins to an RNA oligonucleotide containing the A-rich Hfq-binding motif present at the translation initiation region from *gluP*. (**b**) Molar ratio of the *A*. *vinelandii* Crc and Hfq proteins needed to form a ribonucleoprotein complex with the RNA containing the A-rich Hfq-binding *gluP* motif. As a control an RNA oligonucleotide lacking an A-rich motif was used (**c**). (**d**) Binding of the *P*. *putida* (Pp) Crc and Hfq proteins to the RNA A-rich Hfq binding motif of *gluP*. RNA and protein-RNA complexes were resolved in a non-denaturing polyacrilamide gel. The concentration of Crc (expressed as monomers) and Hfq (expressed as hexamers) is indicated. Arrows point to the position of free RNA and of the ribonucleoprotein complex (RNP). (**e**) Sequence of the *gluP* mRNA leader region. The underlined sequence corresponds to the RNA oligonucleotide used in the band-shift assays, which contains the A-rich motif. The AUG translation initiation codon is in bold face. The sequence of the oligonucleotide used as control in panel (**c**), named DmpR 6C, is also shown.

*A*. *vinelandii* Crc and Hfq-His protein preparations were used in RNA electrophoretic mobility-shift assays (EMSAs) to investigate their ability to recognize the putative A-rich Hfq-binding motif identified close to the AUG translation start codon of *gluP* mRNA. This analysis was achieved by using a 26 nt end-labelled RNA oligonucleotide containing the *gluP* A-rich Hfq-binding target. Consistent with previous reports, the *A*. *vinelandii* Crc protein alone was unable to bind to this A-rich motif (Fig. 7a), while Hfq-His could bind to the RNA oligonucleotide forming a weak complex that dissociated during electrophoresis. However, when both proteins were present, a clear ribonucleoprotein complex was formed (Fig. 7a). Based on the intensity of the shifted band, titration assays showed that efficient formation of the ribonucleoprotein complex required approximately equimolar amounts of Hfq and Crc (considering Hfq as a hexamer) (Fig. 7b). Complex formation was favoured when Hfq was in excess relative to Crc, as has been shown for *alkS* mRNA in *P*. *putida* ^13^. This stable complex was not observed with the control RNA lacking an A-rich Hfq-binding motif (Fig. 7c). Based on these results, we concluded that Crc-Hfq proteins form a complex with the *gluP* leader at the A-rich Hfq-binding motif.

### The *P. putida* Hfq and Crc proteins can bind to the leader of *A. vinelandii gluP*

Because we were unable to obtain an *A*. *vinelandii crc* mutant derivative, we analysed whether the *P*. *putida* Crc and Hfq proteins can regulate the expression of the *A*. *vinelandii gluP* gene. As a first approach, we explored whether the *P*. *putida* proteins Hfq and Crc could form a complex with the leader of *gluP* mRNA *in vitro*. Band-shift assays were conducted using purified *P*. *putida* Hfq-His and Crc proteins extracted from an Hfq-null *E*. *coli* expression strain and the 26 nt end-labelled *gluP* RNA oligonucleotide used in the preceding section. When applied independently, neither the *P*. *putida* Crc nor the Hfq protein could bind to the *gluP* A-rich Hfq-binding motif at the tested concentrations (Fig. 7d). However, when both proteins were present, a stable ribonucleoprotein complex was formed. This result implies that the *P*. *putida* Hfq and Crc proteins are able to recognize and form a complex with the A-rich motif of the *gluP* mRNA *in vitro*.

### Effect of the *P. putida* Crc protein on the translation of *gluP* mRNA

**Figure 8:**
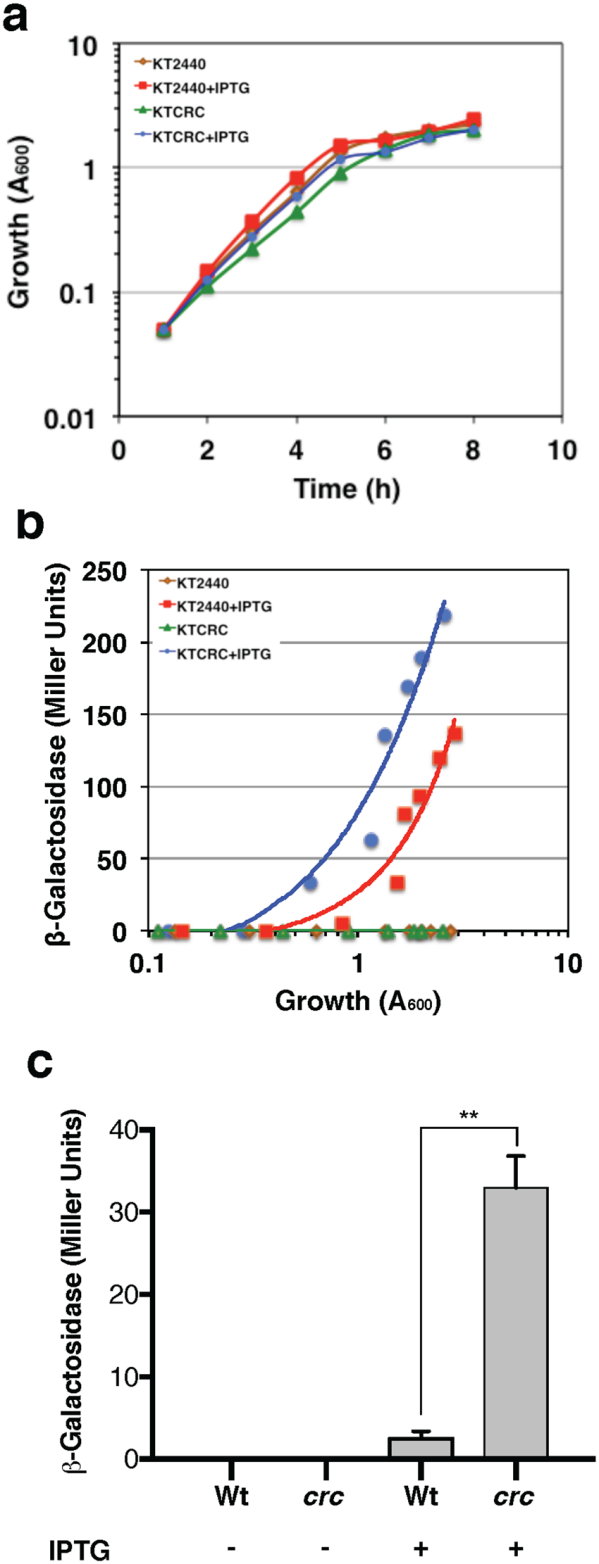
Effect of the Crc protein on the expression of a *gluP’-lacZ* translational fusion in *P*. *putida*. The strains used were KT2440 (wild type) and KTCRC (*crc*::*tet*) carrying plasmid pEQ424P (*gluP’-lacZ)*. Cells were grown in LB medium and where indicated, expression of the *gluP’-lacZ* translational fusion was induced from the *Ptrc* promoter by addition of 0.5 mM IPTG. (**a**) Growth kinetic (measured as the turbidity at 600 nm) of each strain. (**b**) Activity of ß-galactosidase as a function of cell growth. (**c**) ß-galactosidase activity values of the *gluP’-lacZ* translational fusion observed at a turbidity of 0.6 (mid-exponential phase) for KT2440 or KTCRC strain. Three independent assays were performed, and a representative one is shown. Significant difference was analyzed by *t*-test. Statistical significance is indicated (**p < 0.01).

To analyse the effect of the *P*. *putida* Crc protein on *gluP* translation, a post-transcriptional reporter fusion was constructed as previously reported^31^. This reporter fusion consists of a *gluP’-lacZ* translational fusion (60 nt of *gluP* relative to the ATG translation initiation site, which includes the A-rich Hfq-binding motif and the first 8 codons of *gluP*, fused in frame to *lacZ*) cloned into plasmid pSEVA424. The *gluP’-lacZ* sequence is transcribed from the heterologous *Ptrc* promoter of the vector, which can be activated by addition of IPTG. The generated plasmid, named pEQ424P, was introduced into *P*. *putida* strains KT2440 and KTCRC (a *crc::tet* derivative of KT2440). In cells growing in LB medium, ß-galactosidase activity was not detected when no IPTG was added to the growth medium and increased strongly in the presence of 0.5 mM IPTG (Fig. 8b). Interestingly, at the mid-exponential phase, ß-galactosidase activity in the presence of IPTG was approximately 6-fold higher in the *crc* genetic background than in the wild type strain (Fig. 8c). These results indicate that the Crc-Hfq proteins from *P*. *putida* recognize the *gluP* A-rich Hfq-binding motif reducing translation in a Crc-dependent manner.

## Discussion

One of the main characteristics of the free-living *Azotobacter* genus is its capacity for fixing nitrogen in aerobiosis^5^. In a previous study, the preference for acetate assimilation over glucose was shown in *A*. *vinelandii* cultivated under nitrogen fixing conditions^8, 9^. In this study, we reported the characterization of the regulatory systems CbrA/CbrB and Crc/Hfq in controlling the process of CCR, specifically the catabolic repression of glucose consumption, under diazotrophic conditions. Similar to members of the *Pseudomonas* genus, we found that the two-component system CbrA/CbrB is at the head of the regulatory cascade controlling CCR. Inactivation of the gene encoding CbrA diminished expression of the sRNAs CrcZ and CrcY at least 4-fold under standard laboratory conditions, i.e., minimal Burk’s medium amended with 2% sucrose (Fig. 3). Consistent with this result, putative RpoN promoters and sites recognized by the CbrA cognate RR CbrB were identified in the regulatory region of both *crcZ* and *crcY* genes. Our results revealed low expression levels of these sRNAs in the strain lacking CbrA. These results could be attributed to non-specific phosphorylation of the CbrA-cognate RR CbrB. However, the existence of alternative CbrA/CbrB-independent promoters driving *crcZ* and *crcY* expression cannot be ruled out. Putative sequences for σ^70^ promoters were also identified in the regulatory regions of both *crcZ* and *crcY* (Fig. 1 and Supplementary Fig. S2), but their functionality and physiological relevance need to be further investigated.

RNA EMSAs demonstrated that CrcZ from *A*. *vinelandii* formed a stable ribonucleoprotein complex with *A*. *vinelandii* proteins Hfq and Crc, as has been shown in *P*. *putida* ^13^. Hfq was capable of recognizing the CrcZ A-rich Hfq-binding motifs, but the presence of Crc changed the electrophoretic mobility of the shifted band and enhanced the stability of the complex (Fig. 4b and c). This result implies that the sRNA CrcZ (and possibly CrcY) antagonizes the repressing activity of Hfq and Crc and prevents them from controlling their target genes, as it has been proposed to occur in several *Pseudomonas* species. The HK CbrA was necessary for the assimilation of glucose either as the sole carbon source or when mixed with acetate (Fig. 2). This result was attributed to low expression levels of the CrcZ and CrcY sRNAs in the *cbrA* genetic background that in turn allowed the Hfq-Crc repressing activity. Indeed, we found that over-expression of the *A*. *vinelandii* Crc protein from an inducible promoter in the wild type strain AEIV arrested cell growth at the mid-log phase when the strain was cultured in the presence of glucose (Fig. 5a). This result further indicates the ability of Crc to repress glucose assimilation.

We found that *A*. *vinelandii* imports glucose using a GluP transporter, a protein that is absent in most *Pseudomonas* spp. GluP was essential for glucose consumption in *A*. *vinelandii*, whilst expression of *gluP* in an *E*. *coli* mutant (with inactivated or deleted genes encoding the major glucose transporters) restored the mutant’s ability to grow in the presence of glucose (Fig. 6c). Similarly, the GluP homologue from *B*. *abortus*, a glucose and galactose transporter, was functional in *E*. *coli* ^30^. *A*. *vinelandii* GluP belongs to the FGHS family of H^+^-coupled symporters. This finding is consistent with an early report describing active transport of D-glucose by membrane vesicles prepared from *A*. *vinelandii* cells^7^. The transport of glucose was coupled to the oxidation of L-malate and was induced by growth of the cells on D-glucose but arrested in cells grown on acetate. The presence of GluP in *A*. *vinelandii*, rather than the GtsABC sugar ABC transporter commonly found in *Pseudomonas* species, might be due to the intrinsic physiological and metabolic characteristics of this bacterium. A global comparison of transport capabilities among several bacterial species suggested that the preponderance of proton motive force-driven transporters, versus ATP-driven permeases, seems to be explained by the type of energy source most readily generated by the organism^32^. *A*. *vinelandii* exhibits a high respiratory rate which is needed to protect the nitrogenase enzyme from inactivation by oxygen during aerobic nitrogen fixation, a process that has not been reported in *Pseudomonas* species^1, 2^. Therefore, the proton motive force generated by the highly active respiratory electron-transport chain in *A*. *vinelandii* can explain the presence of the GluP H^+^-dependent symporter.

Although the Hfq-Crc system in *Pseudomonas* spp. is known to exert a posttranscriptional effect it has been reported that the transcript abundance of target genes could be also altered, presumably reflecting the effects of translational control on mRNA stability^20^. Interestingly, *gluP* mRNA levels were reduced in the presence of acetate and increased 19-fold during glucose assimilation. Moreover, the *gluP* mRNA levels were reduced by Crc over-expression or in the absence of the HK CbrA, suggesting that *gluP* could be one of the Crc-Hfq targets during CCR control. Indeed, we found that Crc and Hfq proteins from either *A*. *vinelandii* or *P*. *putida* cooperated to form a stable ribonucleoprotein complex with the *gluP* RNA A-rich Hfq-binding motif (Fig. 7a and d). Moreover, the presence of the *gluP* A-rich Hfq-binding motif at the leader region of the *lacZ* reporter gene inhibited translation in *P*. *putida* in a Crc-dependent manner. These results imply that the Crc-Hfq proteins from *A*. *vinelandii* and *P*. *putida* are functionally interchangeable which is further reinforced by the fact that these two proteins are highly conserved in these organisms (Supplementary Fig. S5).

The *A*. *vinelandii eda-1* gene, encoding a KDPG-aldolase enzyme of the ED pathway for glucose assimilation, is predicted to have an A-rich Hfq-binding motif (Supplementary Table S1). EMSAs showed that the Crc-Hfq proteins from either *A*. *vinelandii* or *P*. *putida* could recognize this motif (Supplementary Fig. S6), implying that the *eda-1* gene is a target of these proteins. The genome of *A*. *vinelandii* has been shown to be abundant in highly similar homologues among carbohydrate metabolism genes^33^. The *eda-1*paralogue *eda-2* (Avin15720) shares 81% identity with *eda-1* but it lacks apparent A-rich Hfq-binding motifs. Taken together these results suggest that glucose uptake may constitute a key step during the CCR process for this carbohydrate in *A*. *vinelandii*. However, other genes involved in the assimilation of glucose are also controlled by Crc/Hfq, a multi-tier strategy that has also been observed in *P*. *putida* ^34^.

The *cbrA*::miniTn*5* mutant was able to utilise sucrose as the sole carbon source. Growth in this carbon source, however, was compromised as the *cbrA*::miniTn*5* mutant reached three-fold less biomass than the parental strain (Fig. 2a). Given the reiteration of genes for the metabolism of glucose explained above, we speculated that the uptake of this disaccharide might be subjected to CCR control. In agreement with this idea a putative A-rich Hfq-binding motif (AAAAACAA) at the leader region of *lacY* (Avin51800; encoding the sucrose permease) was identified. Since growth on sucrose was allowed in the absence of *cbrA* or upon Crc sobre-expression, two conditions that clearly inhibited growth on glucose, it is likely that *lacY* is not subjected to a strong CCR control, as is the case for the glucose transporter GluP.

In conclusion, in this study we presented evidence indicating that glucose transport in *A*. *vinelandii* occurs through a GluP transporter, rather than through the GtsABC sugar transporter present in most *Pseudomonas* spp. However, we found that *gluP* expression is under the control of the CbrA/CbrB and Crc/Hfq systems, as it occurs with the *gtsABC* genes in *Pseudomonas* spp.^21, 35, 36^. In fact, we showed that during diazotrophic growth the *A*. *vinelandii* CbrA/CbrB and Crc/Hfq systems operate in a similar manner and they are functionally conserved with respect to *P*. *putida* and other *Pseudomonas* species in regulating the CCR process. This finding reinforces the importance of these regulatory systems in the metabolism of members of the *Pseudomonadaceae* family.

## Methods

### Bacterial strains and culture conditions

Bacterial strains, plasmids and oligonucleotides used in this study are listed in Tables S2, S3 and S4, respectively. *A*. *vinelandii* was grown in minimal Burk’s -sucrose medium as previously reported^37^. *E*. *coli* DH5α^38^ and EC6779 strains^12^ were grown on Luria-Bertani (LB) medium at 37 °C^39^. The WHIPC strain was grown in Mineral M9 medium supplemented with 2.5 gl^−1^ of glucose. *P*. *putida* wild type strain KT2440^40^ and its derivative KTCRC were grown in LB as previously reported^34^. When needed, the final antibiotic concentrations (in µgml^−1^) used for *A*. *vinelandii* and *E*. *coli* were as follows: kanamycin (Km) 1.5 and 10; spectinomycin (Sp), 100 and 100; tetracycline (Tc) 10 and 10. *A*. *vinelandii* transformation was carried out as previously described^41^. The Supplementary Methods summarize general nucleic acid procedures.

### Construction of plasmids pSRK-crc and pSRK-*gluP*

*crc* ORF flanked with SacI and HindIII restriction sites was PCR amplified using primers SPcrc-F (SacI) and crc-R (HindIII). This fragment (1053 bp) was ligated into corresponding sites of pSRKKm^26^, generating pSRK-crc. *gluP* ORF was PCR amplified with primers gluP-F and gluP-R and was cloned into vector pJET 1.2 (Thermo Fisher Scientific), which produced plasmid pAH01. A 1.9 kb BglII-XhoI DNA fragment containing *gluP* was released from pAH01 and was cloned into the corresponding sites of vector pSRKKm, which produced plasmid pSRK-gluP. Plasmids derived from pSRKKm were used to express the cloned gene from an isopropyl-β-D-thiogalactopyranoside (IPTG) inducible *lac* promoter.

### Construction of plasmid pEQ424P

To generate a translational fusion of the *gluP* leader region with *lacZ*, an EcoRI-BamHI fragment of 670 bp (−630 to +28 nt of the *gluP* gene) was PCR amplified using primers gluPF-EcoR1 and gluPR-Bam and was cloned between the corresponding sites of plasmid pUJ9^42^. pUJ9 contains a promoterless *lacZ* gene, and generates plasmid pUJEQP. A post-transcriptional reporter fusion (P*trc*-*gluP’-lacZ*) was constructed in plasmid pSEVA424^43^, in which the *gluP’-lacZ* translational fusion could be transcribed from the P*trc* promoter of the vector upon addition of IPTG. To this end, the *gluP’-lacZ* was PCR-amplified with primers LgluP-FwEcoRI and LacZ-RevHindIII containing restriction sites for EcoRI and HindIII, respectively, and using plasmid pUJEQP as a template. The amplified fragment was EcoRI-HindIII double digested and cloned into the corresponding restriction sites of plasmid pSEVA424, producing plasmid pEQ424P. The correct construction was verified by DNA sequencing. Plasmid pEQ424P was introduced by electroporation into *P*. *putida* wild type strain KT2440^40^ and its derivative KTCRC that carries an inactivated *crc*::*tet* allele^34^.

### Generation of mutant GG15 (*cbrA*::miniTn*5*)

Construction of a random miniTn*5* mutant bank derived from strain AEIV with the miniTn*5*SSgusA40 (Sp^r^) transposon^44^ was previously reported^45^. Mutant GG15 was identified in this mutant bank due to its highly mucoid phenotype on plates of Burk’s-sucrose medium. The miniTn*5* lacks PstI restriction sites. Therefore, the chromosomal region interrupted by the miniTn*5* was cloned as a PstI fragment into vector pBluescript *KS* ^*+*^ (Stratagene), producing plasmid pGG15. Nucleotide sequencing across the transposon insertion junction, using primers Tn5O and Tn5I^45^ and plasmid pGG15 as DNA template was conducted and showed that the *cbrA* gene was disrupted in mutant GG15.

### Construction of *A. vinelandii* mutants

*A*. *vinelandii* was transformed with linear DNA carrying the desired mutation to ensure double reciprocal recombination and allelic exchange. Transformants were selected on Burk’s-sucrose medium amended with the corresponding antibiotic. Gene inactivation was confirmed by PCR analysis. For mutant AEalgD, AEIV cells were transformed with plasmid pJGD (*algD*::Km) linearized with PstI. The resulting Km^r^ mutant was named AEalgD, and the corresponding gene inactivation was confirmed by PCR analysis using oligonucleotides algDF and algDR. For mutant AH1, competent *A*. *vinelandii* cells of mutant AEalgD were transformed with plasmid pGG15 (*cbrA*::miniTn5). Transformants Sp^r^ were selected, and the presence of the *cbrA*::miniTn5 mutation and the absence of wild type copies of *cbrA* were verified by PCR amplification using oligonucleotides F-1(cbrA) and R-1(cbrA). For the construction of mutant EQR02 (*cbrA*::Sp), plasmid pJGEY2 (*cbrA*::Sp) was linearized with EcoRI and was used to transform *A*. *vinelandii* AEIV cells, and double recombinants Sp^r^ were selected. The resulting *cbrA*::Sp mutant was named EQR02. PCR amplification of the *cbrA locus* with oligonucleotides F-1(cbrA) and R-1(cbrA), confirmed the presence of the *cbrA*::Sp mutation and the absence of wild type copies of the *cbrA* gene. Mutant AHI30 (*gluP*::Sp) was constructed by transforming wild type AEIV cells with plasmid pAH03, previously linearized with XhoI, and transformants resistant to Sp were selected. The presence of the *gluP*::Sp mutation and the absence of wild type *gluP* alleles were confirmed by PCR amplification patterns using primer pairs gluP-F and gluP-R. Construction of plasmids pJGD, pJGEY2 and pAH03 is detailed in the Supplementary Methods.

#### Construction of chromosomal *PcrcZ*-*gusA* and *PcrcY*-*gusA* transcriptional fusions

For the construction of strains carrying *PcrcZ*-*gusA* transcriptional fusions, plasmid pEY05 was generated. This plasmid carries a *PcrcZ*-*gusA* transcriptional fusion that can be integrated into the *scrX locus* in the *A*. *vinelandii* chromosome. Construction of plasmid pEY05 is described in the Supplementary Methods. Competent *A*. *vinelandii* cells of strains AEIV and EQR02 (*cbrA*::Sp) were transformed with plasmid pEY05 (*PcrcZ*-*gusA*, Tc^r^) previously linearized with enzyme NdeI. AEIV and EQR02 Tc^r^ derivatives were iso lated and confirmed, by PCR amplification patterns to carry the *PcrcZ*-*gusA* construction; the resulting strains were named AE-Zgus and CbrA-Zgus, respectively. For the construction of strains carrying *PcrcY*-*gusA* transcriptional fusions plasmid pGJ112 was generated. This plasmid is derived from plasmid pUMATcgusAT^46^ and carries the regulatory region of *crcZ* directing transcription of the *gusA* reporter gene to be integrated into the *melA locus*. Construction of plasmid pGJ112 is described in the Supplementary Methods. Competent *A*. *vinelandii* cells from strains AEIV and EQR02 (*cbrA*::Sp) were transformed with plasmid pGJ112 (Tc^r^) previously linearized with enzyme NdeI. AEIV and EQR02 derivatives Tc^r^ were isolated and confirmed, by PCR amplification patterns to carry the *PcrcY-gusA* construction; the resulting strains were named AE-Ygus and Cbr-Ygus, respectively.

### Construction of the *E. coli* mutant WHIPC

Mutant WHIPC carries mutations in genes encoding the major glucose transporter systems (∆*ptsHIcrr*, ∆*mglABC*::FRT-Cm-FRT, *galP*::FRT) and was constructed from mutant WHIP^47^ which lacks the galactose:H^+^ symporter GalP transporter and genes encoding the general components of the phosphoenol pyruvate:sugar phosphotransferase (Pts) system (*galP*::FRT; ∆*ptsHIcrr*). The *mglABC* operon was inactivated in mutant WHIP as previously described^48^, employing PCR products amplified with primers mglABCDtF and mglABCDtR and plasmid pKD3 as the DNA template^47^. Verification of the chromosomal *mglABC* deletion in mutant WHIPC was accomplished by PCR using primers mglABF and mglABR as previously reported^47^.

### Quantitative real time reverse transcription (qRT-PCR)

*A*. *vinelandii* strains were cultured in Burk’s minimal medium supplemented with the indicated carbon source. Details of total RNA extraction, the primers design, cDNA synthesis and qRT-PCR amplification conditions are reported in the Supplementary Methods. The sequences of the primer pairs used for the genes *gluP* (gluP qPCR Fw and gluP qPCR Rv) and 16 s (16 S Fw and 16 S Rv) are listed in Table S4. As in previous studies^49^, 16 s rRNA (Avin55110) was used as internal control in the same sample to normalize the results obtained, as its expression was constant under the tested conditions. The quantification technique used to analyze the generated data was the 2-∆,∆CT method reported previously^50^.

### RNA band-shift assays

*A*. *vinelandii* Crc and Hfq-His proteins were expressed and purified as described in the Supplementary Methods. RNA oligonucleotides containing the A-rich Hfq-binding motifs of *gluP* and *eda1*, were synthesized by Sigma, labelled with T4 polynucleotide kinase and [γ-^32^P]-ATP and purified through MicroSpin G-25 columns (GE Healthcare). Their sequences are as follows: *gluP*, 5′-**AAGCAACAA**UC**ACAACAA**GAGGAAAU; *eda-1*, 5′-CAUCCAG**AACAACAAA**CCGGCCACUU. CrcZ sRNA was obtained by *in vitro* transcription. To this end, *crcZ* gene flanked with HindIII and EcoRI restriction sites was obtained by PCR using primer pairs crcZHd-Fw and crcZEco-ROK and was ligated into the corresponding sites of pTZ19r (Thermo Fisher Scientific), which generated pEQZIT. The insert was sequenced to ensure that it contained the desired DNA fragment. Radioactively labelled CrcZ was obtained as previously described^25^. EMSAs were conducted as described^13^.

#### Analytical methods

Protein levels were determined as previously reported^51^. β-Glucuronidase activity was determined as previously described^52^. One U corresponds to 1 nmol of *o*-nitrophenyl-β-D-glucuronide hydrolysed per min per µg of protein. The β-galactosidase activity of *P*. *putida* cells was determined as follows: an overnight culture of *P*. *putida* was diluted to a final turbidity (A_600_) of 0.05 in fresh LB medium. Where indicated, 0.5 mM IPTG was added to induce transcription from promoter *Ptrc*. Cells were allowed to grow at 30 °C with vigorous aeration, and aliquots were taken at different time points. β-galactosidase activity was measured as previously described^39^ using *o*-nitrophenyl-β-D-galactoside as the substrate. Glucose and acetate quantification were performed by HPLC using an Aminex HPX-87H column (300 9 7.8 mm) (Bio-Rad, Hercules, CA, USA). The eluent used was H_2_SO_4_ (7 mM) at a flow rate of 0.8 mlmin^−1^. Glucose and acetate detection were achieved using a refractive index (RI) detector (Waters 2414 detector). All experiments were conducted at least three times, and the results presented are the averages of independent runs. When required, the Figures show the mean values and standard deviations among biological replicates.

#### Statistical analysis

Statistical analyses were performed using GraphPad Prism (version 7.0) software (GraphPad Software, La Jolla, CA). Statistical significance was determined using two-tailed, unpaired Student’s *t*-test, and a *p*-value ≤ 0.05 was considered significant.

## Electronic supplementary material

Supplementary Information
