# Supplementary material for: Glucose uptake in Azotobacter vinelandii occurs through a GluP transporter that is under the control of the CbrA/CbrB and Hfq-Crc systems
Source: Sci Rep. 2017 Apr 12;7:858. doi: 10.1038/s41598-017-00980-5 (PMC5429807; doi:10.1038/s41598-017-00980-5)
Supplement: Supplementary file 1 — Supplementary Information [file 41598_2017_980_MOESM1_ESM.pdf]

Supplementary Information

Glucose uptake in *Azotobacter vinelandii* occurs through a GluP transporter that is under the control of the CbrA/CbrB and Hfq-Crc systems

Elva Quiroz-Rocha[1], Renata Moreno[2], Armando Hernández-Ortíz[1], Juan Carlos Fragoso-Jiménez[3], Luis Felipe Muriel-Millán[1], Josefina Guzmán[1], Guadalupe Espín[1], Fernando Rojo[2] and Cinthia Núñez[1*]

[1] Departamento de Microbiología Molecular, Instituto de Biotecnología, Universidad Nacional Autónoma de México. Cuernavaca, Morelos, México
[2] Departamento de Biotecnología Microbiana, Centro Nacional de Biotecnología, CSIC, Darwin 3, Cantoblanco, 28049 Madrid, Spain
[3] Departamento de Ingeniería Celular y Biocatálisis, Instituto de Biotecnología, Universidad Nacional Autónoma de México. Cuernavaca, Morelos, México

* cinthia@ibt.unam.mx; Tel. (+52) 777 3290873; Fax (+52) 777 3172388.

Supplementary Methods

Nucleic acid procedures. The *A. vinelandii* genome sequence[1] was used for designing the primers used for PCR amplifications. The high fidelity Phusion DNA polymerase (Thermo Scientific) was used for all PCR amplifications using chromosomal DNA from strain AEIV as template.

Plasmids construction.

pJGD. Plasmid pMSD27, which carries the *algD* gene[2] was partially digested with ScaI enzyme disrupting *algD* in the codon 256; ends were refilled with Klenow enzyme and a Km^r cassette, excised with EcoRV endonuclease from plasmid pBSL99[3], was ligated producing plasmid pJGD (*algD*::Km).

pJGEY2. *cbrA* ORF was PCR amplified using oligonucleotides F-1(cbrA) and R-1(cbrA) and was cloned into pMOS *blue* vector (GE Healthcare) producing plasmid pCN48. An Sp^r cassette derived from plasmid pHP45Ω[4] was inserted into the unique StuI site disrupting the *cbrA* gene. The resulting plasmid, named pJGEY2 (*cbrA*::Sp).

pAH03. A fragment of 1.9 kb was PCR amplified using primers gluP-F and gluP-R and cloned into plasmid pCR2.1-TOPO (Invitrogen) generating plasmid pAH01. The EcoRI *gluP* fragment of 1.945 kb, derived from plasmid pAH01, was subcloned into vector pBluescript KS^+ (Stratagene) producing plasmid pAH02. pAH02 was excised with StyI releasing a *gluP* internal fragment of 600 pb. The StyI cohesive ends were made blunt with the Klenow enzyme and a SmaI fragment carrying a Sp^r cassette released from plasmid pHP45Ω[4] was then ligated, generating plasmid pAH03 (*gluP*::Sp).

pEY05. *scrX* was previously shown to be dispensable for vegetative growth[5]. A region of 3.5 kb containing the gene *scrX* was PCR amplified using primers scrX-F and scrX-R. The resulting product was sub-cloned into vector pCR2.1-TOPO (Invitrogen) generating plasmid pEY01. An inverse PCR was conducted to delete the regulatory and the structural region of *scrX*, by using primers scrXInvF and scrXInv-R. The resultant product was digested with enzyme StuI and ligated to a 5 kb fragment containing the *crcZ* promoter, followed by the *gusA* gene and a Tc^r cassette, generating plasmid pEY05 (*PcrcZ-gusA*). The 5 kb fragment was obtained by PCR amplification using primers pJGgusF and pJGgus-

R and plasmid pEY02 as DNA template. Plasmid pEY02 is a pCN154 derivative (Tc^r), used for the construction of chromosomal transcriptional fusions with *gusA* in *A. vinelandii* strain UW16[6], and carries an XbaI-EcoRI fragment of 500 bp comprising the regulatory region of *crcZ*, amplified by PCR using primers pcrcZFXb and pcrcZRRI.

pGJ112. A fragment of 246 bp, carrying the *crcY* regulatory region, was amplified by PCR using primers crcY-Xb-F and crcY-Pst-R, which include restriction sites for XbaI and PstI endonucleases, respectively. This fragment was subcloned into vector pUMATcgusAT[7] previously cut with XbaI and PstI enzymes generating plasmid pGJ112 (*PcrcY-gusA*).

Expression and purification of *A. vinelandii* Crc protein*:* The Crc protein was purified with no His-tag using the Impact-CN intein system (New England Biolabs), as described[8]. To construct a plasmid specifying the Crc-intein fusion, the *crc* gene was PCR amplified using AEIV chromosomal DNA as template and primers crc-NdeFw and crc-XhoRv, containing a restriction site for NdeI and XhoI enzymes, respectively. The resulting fragment was cloned between the NdeI-XhoI sites of the P_{T7} expression vector pTYB1 (New England Biolabs), generating plasmid pEQEcrc. The amplified DNA segment was sequenced to assure the absence of undesired mutations. Plasmid pEQEcrc was transformed into the *E. coli* EC6779, an Hfq-null derivative of the *E. coli* T7 expression strain BL21(DE3)[9], and overproduction of the Crc-intein fusion was induced by addition of 0.5 mM IPTG to cells grown to mid-log phase (A_{600} of 0.6). After 4 h at 37°C, cells were collected and disrupted by sonication in 20 mM Tris-HCl, pH 8, 500 mM NaCl, 0.1% Triton X-100, 50 µM PMSF. After eliminating cell debris by centrifugation for 30 min at 20 000 × *g* at 4°C, the supernatant was loaded onto a chitin column. The column was extensively washed with wash buffer (250 mM NaCl, 50 mM Tris-HCl, pH 7.5). The Crc-intein fusion was cleaved by an overnight treatment with wash buffer containing 100 mM DTT. The Crc protein was eluted from the column, dialysed against 200 mM NaCl, 20 mM Tris-HCl, pH 8. Protein concentration was determined from BCA (Pierce), and stored at −70°C in 20 mM Tris-HCl, pH 8.0; 200 mM NaCl, 1 mM EDTA; 20% glycerol.

Expression and purification of *A. vinelandii* Hfq-His. The *A. vinelandii hfq* gene carrying in frame fusions to six consecutive histidine codons prior to the termination codon, was synthesized as a 267 bp NdeI-XhoI fragment using primers exphfqFw and exphfqR.

This fragment, having a NdeI site overlapping the ATG initiation codon and a XhoI downstream of the termination codon, was sub-cloned between these sites of the T7 expression vector pET22b (Novagen) to generate pET22::Hfq. Purification of *A. vinelandii* Hfq protein was performed using expression plasmid pET22::Hfq. This plasmid was introduced into EC6779 strain. Hfq-His protein was purified from crude cell lysates of a culture grown to OD_{600} of 0.6 prior to overnight induction with 1 mM IPTG by Ni^{2+}-affinity chromatography using a buffer containing 20 mM Tris-HCl, pH 8.0; 300 mM NaCl, 5 mM imidazole and a protease inhibitor cocktail. The Hfq-His protein was recovered by stepwise elution with the same buffer containing 20 to 250 mM imidazole followed by extensive dialysis of a pool of the most concentrated fractions to remove imidazole. Final preparations were stored at –20°C in storage buffer [20 mM Tris-HCl, pH 8.0; 300 mM NaCl, 1 mM ethylenediaminetetraacetic acid (EDTA); 40% glycerol].

Quantitative real time reverse transcription (qRT-PCR). The cells were collected by centrifugation, and the total RNA was extracted as described[10]. Genomic DNA contamination in the total RNA samples was removed with DNase I (Thermo Scientific). The RNA concentration was measured by 260/280 nm ratio absorbance. RNA integrity was analyzed by agarose gel electrophoresis. The absence of DNA was verified by RT-PCR using primers for *rpoS*. cDNA was synthesized using the Revert Aid TM H First Strand cDNA Synthesis kit (Thermo Fisher Scientific) and a mixture of the specific DNA primers. The primers were designed using the Primer3 program (http://bioinfo.ut.ee/primer3/) with an optimal lenght of 20 bases, and a melting temperature of 60ºC. The cDNA generated was used as template for qRT-PCR assays performed with a Light Cycler 480 II instrument (Roche), using the Maxima TM SYBR Green/ROX qPCR Master Mix (2X) kit (Thermo Scientific). Each primer set was validated by verifying specific single product amplification by melting-curve analyses. Then, the efficiency of PCR was assessed by developing standard curves for each amplicon using dilution series of the cDNA corresponding to the reference sample. cDNAs derived from the experimental and reference samples were amplified using quantities within the linear range of the standard curve. Amplification conditions were 10 min at 95° C, and a two-step cycle at 95° C for 15 s and 60° C for 60 s for a total of 40 cycles. The size of all amplimers was from 95 -110 bp. Three biological

replicates (independent cell cultures) were performed with three technical replicates for each one generating similar results.

Supplementary Tables

Table S1. *A. vinelandii* genes involved in glucose transport and catabolism containing putative A-rich Hfq-binding motifs.

**Sequence**	**Position (rel. to AUG)**	**Gene**	**ID number**	**Description**
ACAACAAGA	-7 to -17	*gluP*	Avin_04150	glucose/galactose transporter protein
AAAGAAAAA	-5 to -13	*zwf-3*	Avin_16620	glucose-6-phosphate 1-dehydrogenase
AAAAAGAACAA	-6 to -16	*zwf-2*	Avin_17630	glucose-6-phosphate 1-dehydrogenase
AACAACAAA	-18 to -26	*eda-1*	Avin_27250	keto-hydroxyglutarate-aldolase/keto-deoxy-phosphogluconate aldolase
AAAAACAACAA	-16 to -26	*kguT*	Avin_26900	2-ketogluconate transporter
AAGAACA	+3 to +9	*kguD*	Avin_26910	2-ketogluconate 6-phosphate reductase
AACAACAA	+1 to -8	*eno*	Avin_38790	phosphopyruvate hydratase (enolase)

Table S2. Bacterial strains used in this study

Name	Genotype/Relevant characteristics	Reference
***A. vinelandii* strains**		
AEIV (also name E strain)	Wild type strain	11
EQR02	AEIV derivative carrying a *cbrA*::Sp mutation. Sp^r	This work
AEalgD	AEIV derivative carrying an *algD*::Km. Unable to produce alginate. Km^r	This work
GG15	AEIV derivative carrying a *cbrA*::miniTn*5* mutation. Highly mucoid. Sp^r	This work
AE-Zgus	AEIV derivative carries a chromosomal *crcZ-gusA* transcriptional fusion. Tc^r	This work
CbrA-Zgus	EQR02 derivative carries a chromosomal *crcZ-gusA* transcriptional fusion. Sp^r, Tc^r	This work
AE-Ygus	AEIV derivative carries a chromosomal *crcY-gusA* transcriptional fusion. Tc^r	This work
CbrA-Ygus	EQR02 derivative carries a chromosomal *crcY-gusA* transcriptional fusion. Sp^r, Tc^r	This work
AH1	GG15 derivative carries an *algD*::Km mutation. Sp^r, Km^r	This work
AHI30	AEIV derivative carries a *gluP*::Sp mutation. Sp^r	This work
***P. putida* strains**		
KT2440	Wild type strain	12
KTCRC	KT2440 *crc* null mutant; Tc^r	13
***E. coli* strains:**		
DH5a	*supE44 DlacU169 hsdR17 recA1 endA1 gyrA96 thi-1 relA1*	14
WHIP	Δ*ptsHIcrr, galP*::FRT	15
WHIPC	Δ*ptsHIcrr,* Δ*mglABC*::FRT-Cm-FRT, *galP*::FRT	This work
EC6779	Hfq null derivative of BL21 (DE3), Cm^r	9

Abbreviations: Cm, chloramphenicol; Tc, tetracycline; Sp, spectinomycin; Km, kanamycin.

Table S3. Plasmids used in this study

Plasmids	Relevant characteristics	Reference
pJET1.2	Cloning vector; Ap^r	Thermo Fisher Scientific
pCR2.1-TOPO	Clonning vector; Ap^r Km^r	Invitrogen
pBluescript *KS*+	Used for subcloning of DNA; Ap^r	Stratagene
pMOS *Blue*	Clonning vector; Ap^r	GE Healthcare
pMSD27	Plasmid containing 5.5 kb of *A. vinelandii* DNA including *algD*. Tc^r, Ap^r, Cm^r.	2
pUMATcgusAT	Vector with the *gusA* gene for transcriptional fusions; Ap^r , Tc^r	7
pCN154	Vector with the *gusA* gene for transcriptional fusions; Ap^r , Tc^r	6
pHP45Ω	Source of the Sp^r cassette	4
pUJ9	*lacZ* promoterless vector, for translational fusions, Ap^r	16
pTZ19r	T7 promoter expression vector, Ap^r	Thermo Fisher Scientific
pSEVA424	IPTG inducible P*trc* expression plasmid; Sm^r	17
pET22b	T7 expression vector, C-terminal histidine tag; Ap^r	NOVAGEN
pTYB1	T7 expression vector for in-frame fusion with an intein tag; Ap^r	New England Biolabs
pKD3	FRT-Cm-FRT, Ap^r	18
pSRKKm	Replicative vector *in A. vinelandii* contains an IPTG inducible promoter. Km^r	19
pCN48	pMOS *Blue* vector derivative carries a 2.8 kb fragment containing the *cbrA* gene	This work
pJGD	pMSD27 derivative carries an *algD*::Km mutation	This work
pJGEY2	pCN48 derivative carries a *cbrA*::Sp mutation	This work
pGG15	pBuescript *KS*+ derivative carrying a PstI fragment containing the *cbrA*::miniTn*5* insertion from strain GG15	This work
pEY01	pCR2.1-TOPO derivative, carries a 3.5 kb fragment of *scrX* gene	This work
pEY02	pCN154 derivative, carries a P*crcZ-gusA* transcriptional fusion.	This work
pEY03	pCR2.1-TOPO derivative, carries the *crcZ locus* of 1.8 kb	This work
pEY05	pEY01 derivative, carries a P*crcZ-gusA* transcriptional fusion	This work
pGJ112	pUMATcgusAT derivative, carries a 0.246 kb fragment of the *crcY* regulatory region	This work
pAH01	pCR2.1-TOPO derivative, carries a 1.9 kb fragment of gene *gluP*	This work
pAH02	pBluescript *KS*+ derivative, carries an EcoRI *gluP* fragment of 1.945 kb, from plasmid pAH01	This work
pAH03	pAH02 derivarive carrying an insertion of a Sp^r cassette within the *gluP* gene	This work

pSRK-crc	pSRKKm derivative carries a copy of the *crc* gene	This work
pSRK-gluP	pSRKKm derivative carries a copy of the *gluP* gene	This work
pUJEQP	pUJ9 derivative; carries a 0.67 kb fragment of the *A. vinelandii gluP* promoter	This work
pEQ424P	pSEVA424 derivative; carries a *gluP-lacZ* translational fusion under the control of the IPTG inducible promotor	This work
pET22::Hfq	pET22b derivative; carries the *A. vinelandii hfq* gene	This work
pEQEcrc	pTYB1 derivative; carries the *A. vinelandii crc* gene	This work
pEQZIT	pTZ19r derivative; carries the *A. vinelandii crcZ* gene	This work

Abbreviations: Cm, chloramphenicol; Tc, tetracyclin; Sm, streptomycin; Ap, ampicillin; Sp, spectinomycin; Km, kanamycin.

Table S4. Sequences of the primers used in this study.

Primer Name	Nucleotide sequence (5′–3′)
UcrcZ-F2	CAG TTC CGT GAG GAC CTG
DcrcZ-R	CAG TTC CGT GAG GAC CTG
scrX-F	GAG CTC CGA TGA CGA TCG CTG GCA AC
scrX-R	GAG CTC GCG TTC TCA GAT GGC TGG TC
scrXInvF	AGGCCTCTATGTAGGTCCTCGCTTG
scrXInv-R	AGGCCTTAGGGCAACCGTGCCAGAC
pJGgusF	GACCAGTACGTTTCGGTTCTG
pJGgus-R	CCCTTGAAAGACTCCAGGAAG
pcrcZFXb	TCTAGAGACCTGGAGGACGATGATTTC
pcrcZRRI	GAATTCATTGTGGGTGGTACGTCTTG
gluP-F	CAT GTG GAT CGA CTC AGG AG
gluP-R	CCA GGC ATT CGG TAT AGA AG
SPcrc-F	AGGCCTCTATGTAGGTCCTCGCTTG
crc-R	GAGCTCGCATCCTGATGATGCTCTGC
crcY-Pst-R	CTGCAGTTGTTGTTCTTGAGATACCG
crcY-Xb-F	TCTAGACGGTAACAAACGGACGTTAC
mglABCDtF	AGC ATT TAT CTC AAG CAC TAC CCT GCA TAA GAA AAA CCG GAG ATA CCA TGG TGT AGG CTG GAG CTG CTT C
mglABCDtR	TTT ATG ACC GAA TGC GGA CCA CAT TCA CAT CAT TTC TTA CGC GCG TAT TTA TGG GAA TTA GCC ATG GTC C
mglABF	GCT TCG GCG TTC AGT AAC AC
mglABR	TAT GAC CGA ATG CGG ACC AC
exphfqFw	ATC CAT ATG TCA AAA GGG CAT TCG
exphfqRv	ATC CTC GAG GGC GTT GCC GGG TTC GGG
crc-Nde Fw	TTT TGG GGG CCA CAT ATG CGG
crc-Xho Rv	ATC CTC GAG CAG GCT CAG CAG CCA GTC G
crcZHd-Fw	GGA AAG CTT CGT ACA ACA ACA ATA ACA
crcZEco-ROK	ACCGAATTCGAACGCAGAAAGGGAGCC
gluP F EcoR1	TTT TTG AAT TCT GAA ACG ATT CAT CAA TA
gluP R Bam	TTTTTGGATCCTCTACCGTCGTCATCGTG
LgluP-FwEcoRI	TTATTGAATTCGGCCTCTCCTTGAAGCAA
LacZ-RevHindIII	TTATTAAGCTTTTATTTTTGACACCAGACCA
F-1(cbrA)	GCA CCT ACC AAC TGT CGT CC
R-1(cbrA)	GCT GAT GAT CAG GTC GAA GC
algDF	CGATCAAGGACTACAACTTC
algDR	TTGCTGTCGA AGATGCTCAG
16S Fw	ACCGCATCCAAAACTACTGG
16S Rv	GCCACTGGTGTTCCTTCCTA
gluP qPCR Fw	GCGATTGAGCCTGTACGT TT
gluP qPCR Rv	CTGAGGCTGAACAGGTCCTT

Supplementary Figures

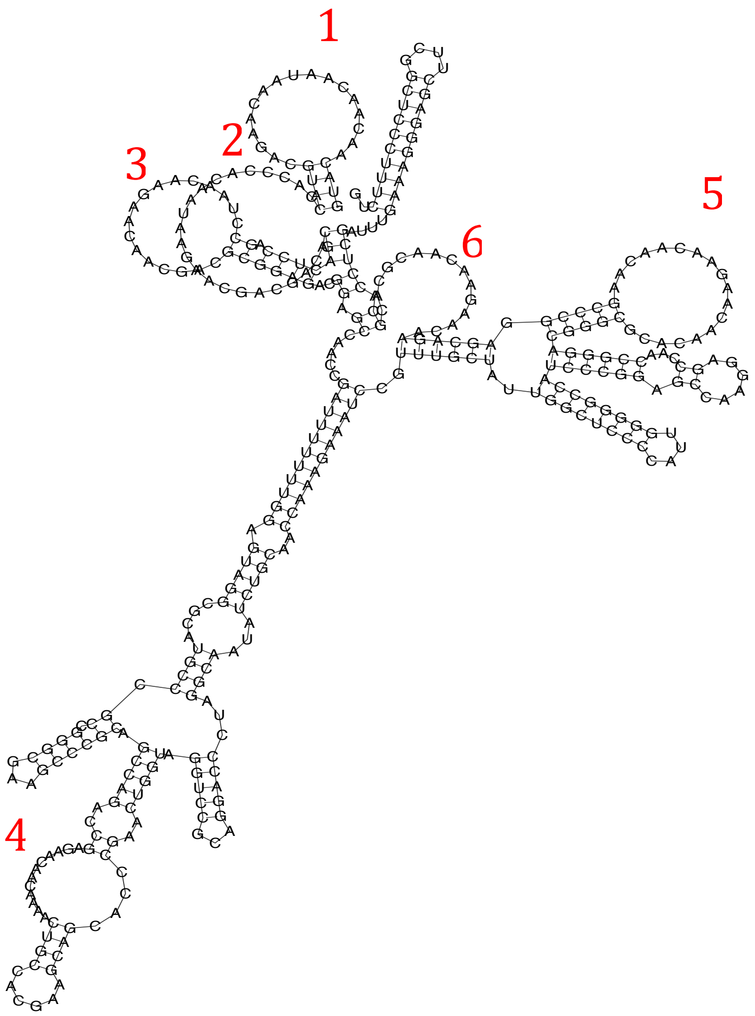

Figure S1. Predicted secondary structure of the *A. vinelandii* small RNA CrcZ. It was conducted using the RNAfold algorithm (http:/ rna.tbi.univie.ac.at/cgi-bin/RNAfold.cgi). The predicted A-rich Hfq-binding motifs 1 to 6 are indicated.

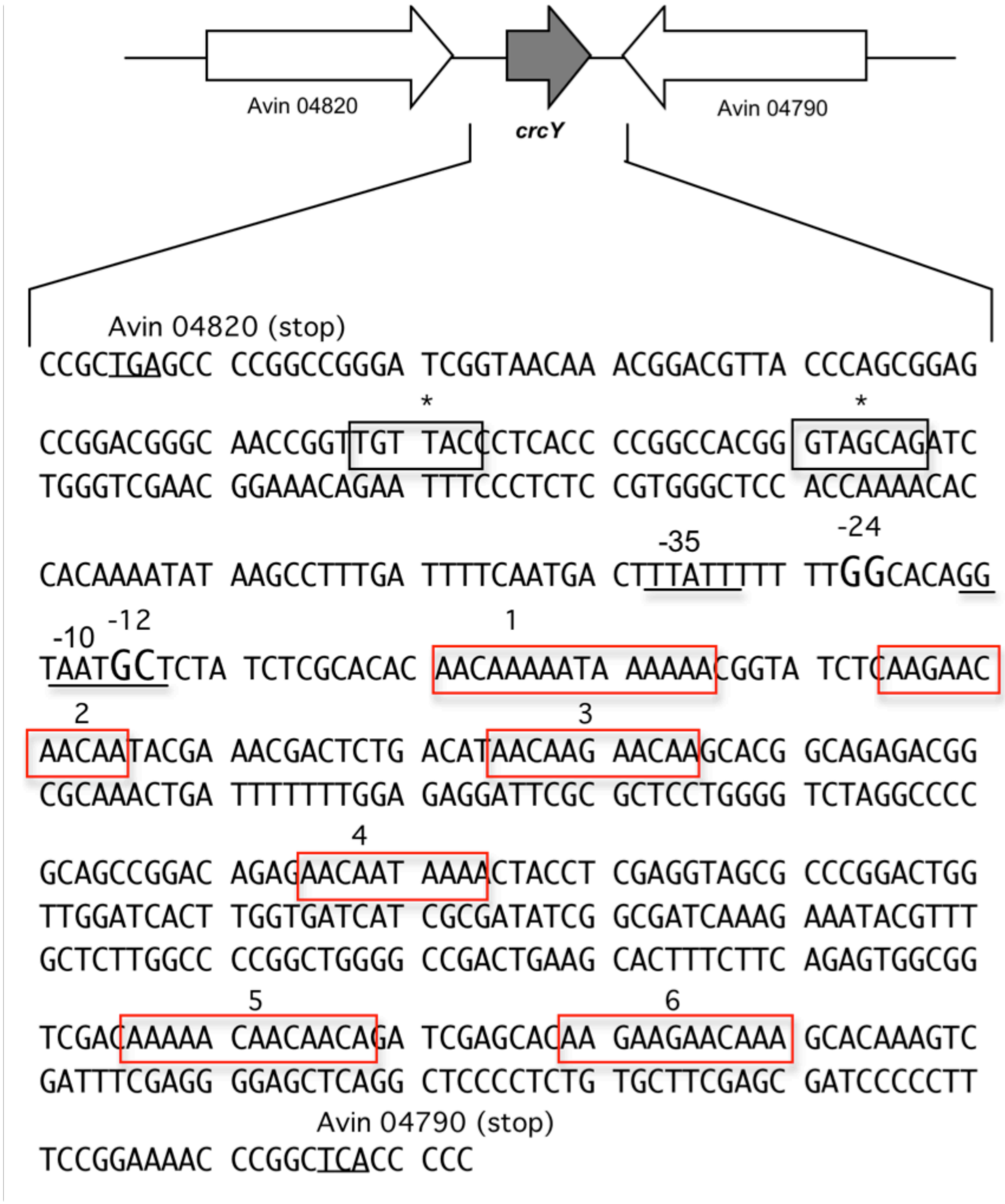

Figure S2. Genomic context and nucleotide sequence of the *A. vinelandii crcY* gene. The six CrcY A-rich Hfq-binding motifs are indicated by red boxes. The -12 and -24 regions of the RpoN predicted promoter as well as putative sequences recognized by CbrB (asterisks) are indicated. The predicted -10 and -35 regions of an RpoD promoter are underlined.

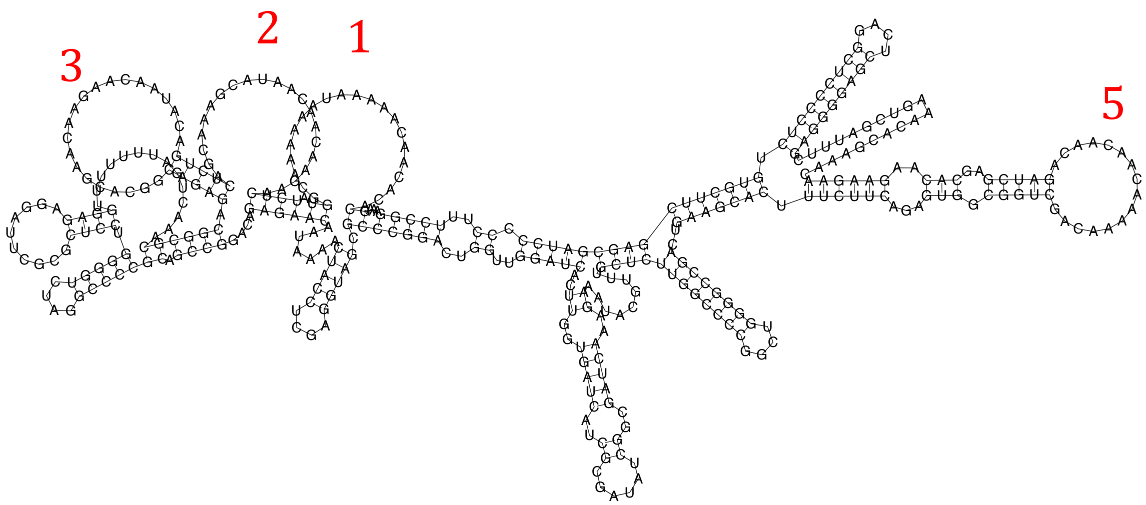

Figure S3. Predicted secondary structure of the *A. vinelandii* sRNA CrcY. It was conducted using the RNAfold algorithm (http:/ rna.tbi.univie.ac.at/cgi-bin/RNAfold.cgi). The predicted A-rich Hfq-binding motifs are indicated by red numbers.

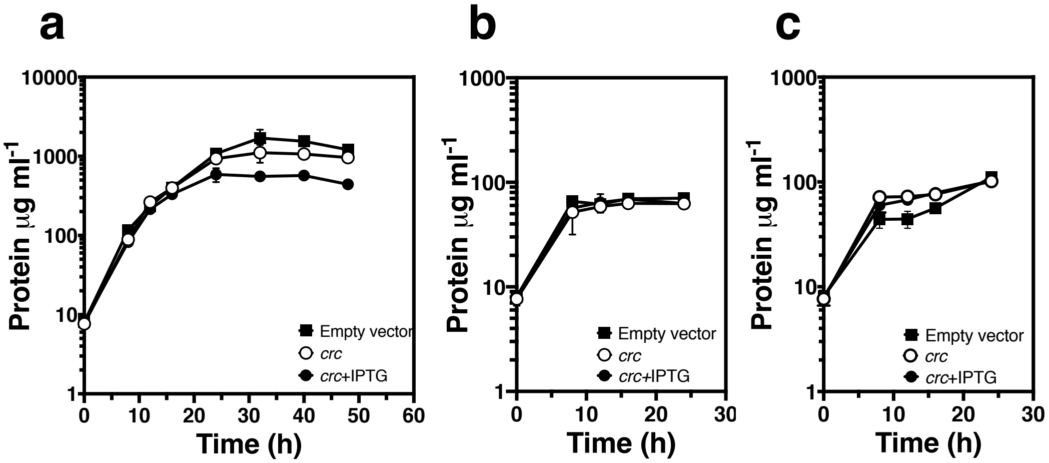

Figure S4. Effect of Crc over-expression on *A. vinelandii* growth. Growth kinetics of the wild type *A. vinelandii* AEIV strain, harbouring the empty vector pSRK-Km (■) or the pSRK-crc (crc^+) vector, in the presence (●) or absence (○) of 1 mM IPTG. Cultures were developed in Burk's minimal medium supplemented with 30 mM of sucrose (a), acetate (b) or succinate (c) as the sole carbon source and 1.5 μgml^{-1} of kanamycin as a selection marker. 25 ml of Burk's medium supplemented with 30 mM sucrose were cultured for 18 h; cells were harvested by centrifugation, washed with phosphate buffer 10 mM pH 7.2 and resuspended in the same solution. 400 μg of these cells were used to inoculate 50 ml of the culture medium and samples were collected at the indicated times for protein quantification. The results represent the averages of the results of three independent experiments, and error bars depict standard deviations.

```
E. coli        MAKGQSLQDPFLNALRRERVPVSIYLVNGIKLQGQIESFDQFVILLKNTVSQMVYKHAIS     60
P. putida      MSKGHSLQDPYLNTLRKEKVPVSIYLVNGIKLQGSIESFDQFVVLLKNTVSQMVYKHAIS     60
A. vinelandii  MSKGHSLQDPYLNTLRKERVPVSIYLVNGIKLQGQIESFDQFVILLKNTVSQMVYKHAIS     60
               *:**:*****:**:**:*:***************.********:****************

E. coli        TVVPSRPVSHHSNNAGGGTSSNYHHGSSAQNTSAQQDSEETE       102
P. putida      TVVPARPVRLPSPTDSEHGDSEP--GNA--------------       86
A. vinelandii  TVVPSRPVRLPTASEGE--QPEP--GNA--------------       84
               ****:***   : . .   . :   *.:
```

Figure S5. Alignment of Hfq proteins from *E. coli*, *P. putida* and *A. vinelandii*. It was conducted using the Clustal Omega multiple sequence alignment program (https://www.ebi.ac.uk/Tools/msa/clustalo/). UniProtKB aligned sequences are *E. coli* P0A6X3; *P. putida*, Q88DD3; *A. vinelandii*, C1DLQ2. Consensus is indicated below each amino acid residue by symbols: asterisk, conserved residue; colon, residues with strongly similar properties; period, residues with weakly similar properties; no symbol, no conservation of properties. Amino acid residue Y25 involved in recognition of the A-rich Hfq-binding motif of *alkS* mRNA in *P. putida*[8] is indicated by a triangle.

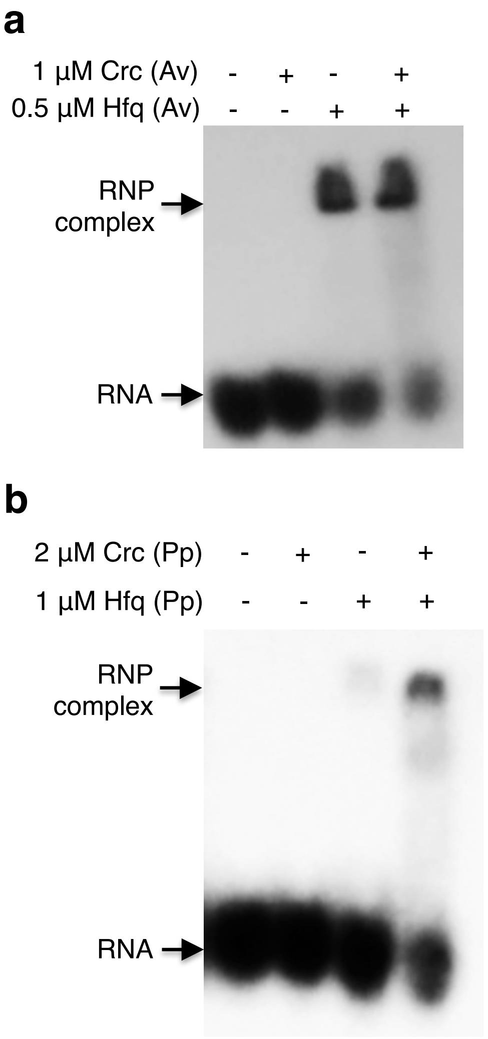

c

eda-1 (Avin 27250)

5`-UGAACAGCAUCCAGAACAACAAACCGGCCACUUC
CCAGAGCGUGCCGAGC**AUG**GCCGACAAGGUCGCCC

Figure S6. Hfq-Crc proteins form a complex with the *eda-1* RNA A-rich Hfq-binding motif. Binding of Hfq and Crc proteins from *A. vinelandii* (Av) (a), or from *P. putida* (pp) (b), to an RNA oligonucleotide containing the A-rich motif present at the translation initiation regions from *eda-1* gene (Avin 27250). RNA and protein-RNA complexes were resolved in a non-denaturing polyacrilamide gel. The concentration of Crc (expressed as monomers) and Hfq (expressed as hexamers) is indicated. Arrows point to the position of free RNA and of the ribonucleoprotein complex (RNP). (c) Sequence corresponding to the *eda-1* mRNA leader region. The underlined sequence corresponds to the RNA oligonucleotide used in the band-shift assays, which contains the A-rich motif. The AUG translation initiation codon is in bold face.

Supplementary References

1 Setubal, J. C. *et al.* Genome sequence of *Azotobacter vinelandii*, an obligate aerobe specialized to support diverse anaerobic metabolic processes. *J Bacteriol* **191**, 4534-4545, doi:10.1128/JB.00504-09 (2009).

2 Campos, M. *et al.* Characterization of the gene coding for GDP-mannose dehydrogenase (*algD*) from *Azotobacter vinelandii*. *J Bacteriol* **178**, 1793-1799 (1996).

3 Alexeyev, M. F., Shokolenko, I. N. & Croughan, T. P. New mini-Tn5 derivatives for insertion mutagenesis and genetic engineering in gram-negative bacteria. *Can J Microbiol* **41**, 1053-1055 (1995).

4 Fellay, R., Frey, J. & Krisch, H. Interposon mutagenesis of soil and water bacteria: a family of DNA fragments designed for in vitro insertional mutagenesis of gram-negative bacteria. *Gene* **52**, 147-154 (1987).

5 Johnson, D. C., Unciuleac, M. C. & Dean, D. R. Controlled expression and functional analysis of iron-sulfur cluster biosynthetic components within *Azotobacter vinelandii*. *J Bacteriol* **188**, 7551-7561, doi:10.1128/JB.00596-06 (2006).

6 Hernández-Eligio, A. *et al.* RsmA post-transcriptionally controls PhbR expression and polyhydroxybutyrate biosynthesis in *Azotobacter vinelandii*. *Microbiology* **158**, 1953-1963, doi:10.1099/mic.0.059329-0 (2012).

7 Muriel-Millan, L. F. *et al.* The unphosphorylated EIIA(Ntr) protein represses the synthesis of alkylresorcinols in *Azotobacter vinelandii*. *PLoS One* **10**, e0117184, doi:10.1371/journal.pone.0117184 (2015).

8 Moreno, R. *et al.* The Crc and Hfq proteins of *Pseudomonas putida* cooperate in catabolite repression and formation of ribonucleic acid complexes with specific target motifs. *Environ Microbiol* **17**, 105-118, doi:10.1111/1462-2920.12499 (2015).

9 Madhushani, A., Del Peso-Santos, T., Moreno, R., Rojo, F. & Shingler, V. Transcriptional and translational control through the 5'-leader region of the *dmpR* master regulatory gene of phenol metabolism. *Environ Microbiol* **17**, 119-133, doi:10.1111/1462-2920.12511 (2015).

10 Barry, T. *et al.* Rapid mini-preparations of total RNA from bacteria. *Nucleic Acids Res* **20**, 4940 (1992).

11 Larsen, B. & Haug, A. Biosynthesis of alginate. 3. Tritium incorporation with polymannuronic acid 5-epimerase from *Azotobacter vinelandii*. *Carbohydr Res* **20**, 225-232 (1971).

12 Franklin, F. C., Bagdasarian, M., Bagdasarian, M. M. & Timmis, K. N. Molecular and functional analysis of the TOL plasmid pWWO from *Pseudomonas putida* and cloning of genes for the entire regulated aromatic ring meta cleavage pathway. *Proc Natl Acad Sci U S A* **78**, 7458-7462 (1981).

13 Hernández-Arranz, S., Moreno, R. & Rojo, F. The translational repressor Crc controls the *Pseudomonas putida* benzoate and alkane catabolic pathways

using a multi-tier regulation strategy. *Environ Microbiol* **15**, 227-241, doi:10.1111/j.1462-2920.2012.02863.x (2013).

14 Hanahan, D. Studies on transformation of *Escherichia coli* with plasmids. *J Mol Biol* **166**, 557-580 (1983).

15 Fuentes, L. G. *et al.* Modification of glucose import capacity in *Escherichia coli:* physiologic consequences and utility for improving DNA vaccine production. *Microb Cell Fact* **12**, 42, doi:10.1186/1475-2859-12-42 (2013).

16 de Lorenzo, V., Herrero, M., Jakubzik, U. & Timmis, K. N. Mini-Tn5 transposon derivatives for insertion mutagenesis, promoter probing, and chromosomal insertion of cloned DNA in gram-negative eubacteria. *J Bacteriol* **172**, 6568-6572 (1990).

17 Silva-Rocha, R. *et al.* The Standard European Vector Architecture (SEVA): a coherent platform for the analysis and deployment of complex prokaryotic phenotypes. *Nucleic Acids Res* **41**, D666-675, doi:10.1093/nar/gks1119 (2013).

18 Datsenko, K. A. & Wanner, B. L. One-step inactivation of chromosomal genes in *Escherichia coli* K-12 using PCR products. *Proc Natl Acad Sci U S A* **97**, 6640-6645, doi:10.1073/pnas.120163297 (2000).

19 Khan, S. R., Gaines, J., Roop, R. M., 2nd & Farrand, S. K. Broad-host-range expression vectors with tightly regulated promoters and their use to examine the influence of TraR and TraM expression on Ti plasmid quorum sensing. *Appl Environ Microbiol* **74**, 5053-5062, doi:10.1128/AEM.01098-08 (2008).
